# Association of heavy metal mixtures with liver function biomarkers: multi-model analysis identifies cadmium as the primary driver

**DOI:** 10.3389/fpubh.2026.1817191

**Published:** 2026-04-28

**Authors:** Honglong Zhang, Xingwang Zhu, Meng Tian, Tingting Wang, Ruipeng Wang, Mingtong Zhang, Jun Yan, Xun Li

**Affiliations:** 1 Department of Breast and Thyroid Surgery, Union Hospital, Tongji Medical College, Huazhong University of Science and Technology, Wuhan, China; 2 The First School of Clinical Medicine, Lanzhou University, Lanzhou, China; 3 Deyang People's Hospital, Deyang, China; 4 Gansu Provincial Institute of Drug Control, Lanzhou, China; 5 Department of General Surgery, The First Hospital of Lanzhou University, Lanzhou, China; 6 Hepatopancreatobiliary Surgery Institute of Gansu Province, Lanzhou, China; 7 Key Laboratory of Biotherapy and Regenerative Medicine of Gansu Province, Lanzhou, China; 8 Clinical Research Center for General Surgery of Gansu Province, Lanzhou, China

**Keywords:** BKMR, cadmium, heavy metal mixture, liver function biomarkers, Qgcomp, WQS

## Abstract

**Background:**

Evidence regarding the hepatotoxic effects of co-exposure to multiple heavy metals in the general middle-aged and older adults population remains limited. This study aimed to investigate the association between heavy metal mixtures and liver function in the population of Northwest China, with key findings supported using an animal model.

**Methods:**

We conducted a cross-sectional study involving 451 participants from the Dongdagou Xinglong cohort. Concentrations of heavy metals and liver function indices were measured. Multiple linear regression, Bayesian kernel machine regression (BKMR), weighted quantile sum (WQS), and quantile-based g-computation (Qgcomp) regression were employed to evaluate the combined effects of co-exposure to multiple heavy metals on liver function. A sub-chronic cadmium (Cd) exposure rat model was further established to validate population-based findings.

**Results:**

Multiple linear regression analysis revealed that blood Cd was positively correlated with GGT (β = 0.236), TBA (β = 0.162), ALT (β = 0.142) and AST (β = 0.114), while negatively correlated with DBil (β = −0.207), TBil (β = −0.166) and IBil (β = −0.157) (all *P* < 0.05). Similarly, other heavy metals also exhibited significant associations with liver function indicators. BKMR analysis showed that heavy metal mixture exposure was positively associated with ALT, AST, ALP, GGT, CHE, and TBA, but negatively associated with TBil, DBil, and IBil; WQS regression indicated that positive associations between the metal mixture and GGT as well as CHE; and the Qgcomp model demonstrated that the metal mixture was positively associated with ALT, GGT, and TBA, and negatively associated with TBil, DBil, and IBil. Notably, all three statistical models consistently identified Cd as the factor associated with liver function biomarkers. Furthermore, animal experiments provided experimental evidence consistent with the human findings: Cd exposure led to elevated serum GGT and ALP levels and induced histopathological alterations in the liver. Transcriptomic sequencing suggested that hepatic lipid metabolism pathways may be involved in Cd-induced liver injury.

**Conclusions:**

Overall, our study shows that co-exposure to heavy metals is associated with liver function biomarkers in middle-aged and older adults, with Cd identified as the predominant factor associated with liver function biomarkers.

## 1 Introduction

Chronic liver disease is a major global health concern. Accumulating evidence indicates that chronic liver disease is a significant risk factor for both chronic kidney disease and cardiovascular disease (1, 2), and liver decompensation as well as acute and chronic liver failure associated with chronic liver disease are the primary causes of liver-related death worldwide (3). Therefore, determining the risk factors linked to the development of liver disease is crucial. Genetic factors, hepatitis virus infection, excessive alcohol consumption, diabetes, and metabolic disorders are clear risk factors for liver damage (4, 5), but cannot explain the occurrence and development of all chronic liver diseases. In recent years, environmental exposure to heavy metals has been reported to cause abnormal liver function (6, 7). A variety of toxic metals enter the body through occupational or non-occupational exposure and accumulate in the liver, damaging its normal physiological functions (8, 9). Our previous study of residents of northwest China found that single or combined exposure to cadmium (Cd) and lead (Pb) increased serum liver biomarkers, including alanine transaminase (ALT) and γ-glutamyltransferase (GGT), leading to abnormal liver function (10). However, residents may be exposed to various environmental heavy metals and further research on the effects of exposure to heavy metal mixtures on human liver function may be more realistic.

In the real environment, heavy metals often coexist in the form of mixtures in water, soil, air, and food; therefore, people are often exposed to multiple heavy metals through the three pathways: ingestion, inhalation, and dermal contact (11). Previous research has consistently linked heavy metal exposure to serious health consequences, including neurological damage, renal dysfunction, and cancer, and the interactions of heavy metals in the body may have complex effects on human health (12). Nevertheless, more previous studies have only assessed the effects of single metal exposure on liver function; for example, manganese (Mn) exposure is a potential marker for the development of non-alcoholic fatty liver disease (NAFLD) and has positive association with advanced liver fibrosis (13); Long-term exposure to Cd significantly increases liver enzyme levels (14), with a 23-year prospective cohort study suggesting that Cd exposure in young adulthood may increase the prevalence of NAFLD in midlife (15). A variety of novel mixture analysis models, including Bayesian kernel machine regression (BKMR), weighted quantile sum (WQS) regression, and quantile g-computation (Qgcomp), have been applied to the field of environmental epidemics, allowing the investigation of the comprehensive effects of environmental mixture exposure on human health (16). Each mixture modeling approach operates under distinct assumptions and offers unique analytical strengths. For instance, BKMR flexibly accommodates non-linear exposure-response functions and potential interactions among mixture components without imposing *a priori* directional constraints (17); WQS regression constructs a weighted index to estimate a cumulative mixture effect under the assumption of unidirectional associations, thereby facilitating the identification of key contributors (18); Qgcomp extends the WQS framework by relaxing the directional homogeneity assumption, allowing for both positive and negative contributions within the same model (19). Given that each method has inherent strengths and limitations, the application of multiple complementary models can ensure that conclusions are robust to model-specific assumptions. Previous studies have used various statistical models to investigate the association between metal mixtures and cardiovascular diseases, rheumatoid arthritis, lung function, and various cancer risks (20–24). However, only a limited body of studies have investigated the effects of metal mixtures on liver function. For instance, Li et al. (25) applied mixture exposure models to the United States National Health and Nutrition Examination Survey (NHANES) data and identified Cd, Pb, and hydrargyrum (Hg) in blood as the most influential metals within the mixture affecting liver enzyme levels. Given the variations in the specific metal compositions identified across studies, further investigation is warranted.

With the rapid development of modern industry, heavy metal pollution and its associated health risks have become increasingly severe worldwide. Unlike occupational acute exposure, environmental heavy metal pollution is predominantly characterized by long-term, low-dose continuous exposure. These pollutants can enter the human body through contaminated air, water, and food, and gradually accumulate over time (26). The chronic health impairments induced by such exposure have emerged as a major focus of research in the field of environmental health. The Dongdagou watershed in northwestern China, severely contaminated by long-term mining activities, is the largest heavy metal-polluted area in the upper reaches of the Yellow River, providing a typical research setting for investigating the health effects of long-term low-dose heavy metal exposure (27). Based on the established Dongdagou Xinglong (DDGXL) prospective cohort, this study explored the association between blood heavy metal concentrations and liver function damage, as well as the key influencing factors.

Therefore, this study enrolled local residents from the DDGXL cohort as participants, and blood heavy metal concentrations were used as internal exposure biomarkers. A combination of statistical models, including multiple linear regression, BKMR, WQS, and Qgcomp, was applied to comprehensively evaluate the associations between co-exposure to multiple heavy metals and liver function indices, and Cd was identified as the metal most consistently associated with liver function indices. Furthermore, a sub-chronic Cd exposure rat model was established to validate the core findings from the human cohort study, and transcriptome sequencing technology was employed to deeply decipher the potential mechanisms underlying Cd-induced liver injury. The results of this study provide important scientific evidence for formulating targeted protective strategies for liver health.

## 2 Materials and methods

### 2.1 Study population

The DDGXL study employed an environmental exposure design, comparing residents from a pre-defined historically polluted area against those from reference area with minimal industrial influence. Residents in polluted areas were exposed to toxic metals through the consumption of cereals, fruits, and vegetables contaminated with heavy metals, whereas residents in the reference areas were almost not exposed to heavy metals. While we did not directly quantify metals in food, the context of soil pollution in our study area is established, known to cause Cd and Pb accumulation in staple grains (28). Previous studies have also demonstrated that vegetables (e.g., carrots, eggplant) from this region often contain metal concentrations exceeding regulatory limits (29, 30). Consequently, we evaluated internal exposure via blood metal biomonitoring to capture the aggregate exposure from all sources. Residents were recruited through a door-to-door survey. Eligible individuals who met the inclusion criteria were invited to complete a structured questionnaire and undergo a comprehensive health examination at local community health centers. The inclusion criteria were: (1) aged 40–69 years; (2) resident in the study area for at least 10 years; (3) consumption of locally produced staple foods (cereals, vegetables, fruits) as the primary dietary source; (4) voluntary participation and signing of written informed consent. Exclusion criteria were: (1) chronic diseases such as liver disease and infectious diseases; (2) past or present suffering from major diseases including malignant tumors (31). A total of 503 participants were initially recruited for this study. After excluding individuals with missing basic information, insufficient blood samples, or incomplete heavy metal and liver function data, 451 participants (158 males and 293 females) were ultimately included in the final analysis. Among these 451 participants, 318 (70.5%) were from the polluted area and 133 (29.5%) were from the reference area (10). The participant selection process is summarized in Supplementary Figure S1. This study was approved by the Ethics Committee of the First Hospital of Lanzhou University (LDYYLL2020-103), and all participants provided written informed consent. All experiments were performed in accordance with relevant guidelines and regulations.

### 2.2 Assessment of heavy metal exposure

The analysis of metal concentrations in whole blood was performed by the Chongqing prevention and treatment center for occupational diseases. Briefly, 0.2 ml of thawed blood sample was mixed with 0.5 ml of sample diluent (a 3:2 mixture of 65% HNO3 and 30% H_2_O_2_) and subjected to digestion using a microwave digestion system (MARS-5, CEM, USA). The concentrations of metals, Aluminum (Al), Chromium (Cr), Copper (Cu), Zinc (Zn), Arsenic (As), Cd, Pb, in the prepared samples were quantified using ICP-MS (NexION 300X, PerkinElmer, USA). The instrumental operating conditions were as follows: RF power of 1200 W, sampling depth of 5.0 mm, plasma gas flow of 9.0 L/min, auxiliary gas flow of 1.1 L/min, nebulizer gas flow of 0.7 L/min, and cooling water temperature of 20 °C. The measurement was conducted in collision/reaction cell (CRC) mode. Each sample was analyzed in triplicate, and the average value was reported. External calibration standards were prepared by serial dilution of a multi-element stock solution. An internal standard solution containing Rhenium (Re) and Rhodium (Rh) was introduced online to all samples, blanks, and calibrants to correct for instrumental drift and matrix effects. Measurements below the limit of detection (LOD) were imputed as LOD/√2. Further details on the metal measurement methodology can be found in a previously published article (31).

Quality assurance and quality control (QA/QC): To ensure data accuracy and precision, each analytical batch included reagent blanks, duplicate samples, and certified reference materials (CRMs; SERONORM™ Trace Elements Whole Blood, SERO AS, Norway). The recovery rates for the target metals in the CRMs ranged from 96.3% to 103.5%, all within the acceptable limits. The relative standard deviation (RSD) for duplicate samples was < 10%. All calibration standards were verified to meet the sample analysis requirements. Detailed quality control procedures are described in a previous publication (31). The fitted calibration curves, LODs, recovery rates, RSDs, geometric mean, arithmetic mean, and interquartile range for the blood metals are provided in Supplementary Table S1.

### 2.3 Measurement of liver function indices

Serum samples were collected from the subjects and nine liver function indices, including ALT, aspartate transaminase (AST), total bilirubin (TBil), direct bilirubin (DBil), indirect bilirubin (IBil), alkaline phosphatase (ALP), GGT, cholinesterase (CHE), and total bile acids (TBA), were measured using an automated chemical analyzer (Beckman AU5800, Japan). The detailed liver function test results can be found in a previously published article (10). All the liver function indices were higher than the LOD values. All liver function indices were analyzed as continuous variables; no clinical cutoffs were applied to define abnormal liver function.

### 2.4 Covariates

Age, sex, body mass index (BMI), smoking status, alcohol consumption, and tea consumption were considered covariates. Smoking status was divided into “yes” and “no,” and smoking was defined as smoking an average of three cigarettes or more per week; alcohol consumption status was divided into “yes” and “no,” and alcohol consumption was defined as drinking alcohol at least once a week; and tea drinking status is divided into “yes” and “no,” tea drinking was defined as drinking more than one cup of tea per day, and more than 6 months. Detailed information regarding each variable can be found in a previous study (32).

### 2.5 Animal experiments

#### 2.5.1 Sub-chronic Cd exposure rat model establishment

Male Sprague-Dawley rats (body weight: 130–150 g) were purchased from Lanzhou Veterinary Research Institute (Lanzhou, China). All rats were housed in individually ventilated cages with a controlled environment: constant temperature (22 °C ± 2 °C), relative humidity (50% ± 10%), 12 h light/12 h dark cycle. Prior to the formal experiment, the rats were acclimatized for 1 week to adapt to the environmental conditions. For the experiment, 14 rats were randomly divided into two groups (*n* = 7 per group) using a random number table: the control group and the Cd treated group. Cadmium chloride (CdCl_2_) was used as the source of Cd exposure in this experiment. The selection of dose and exposure duration was based on our previous study, which demonstrated that rats in the 20.0 and 40.0 mg/kg body weight groups developed significant liver injury after 12 weeks of continuous gavage (33). To better simulate low-dose environmental exposure in humans, and in consideration of other toxicological studies in rats (34), a Cd dose of 20.0 mg/kg was selected for the present study; the control group received an equal volume of sterile pure water. The treatment was administered via oral gavage daily over a period of 12 consecutive weeks. Body weight was measured at a fixed time every week, and general conditions of rats were observed and recorded daily.

#### 2.5.2 Detection of liver function indices

After anesthesia, the body weight of the rats was accurately measured. After disinfection of the abdominal skin, the abdominal cavity was opened and whole blood was collected from the abdominal aorta into anticoagulant-free centrifuge tubes. After standing at room temperature for 30 min, serum was separated by centrifugation at 3,000 rpm for 15 min at 4 °C. Serum levels of ALP and GGT were determined using an automatic biochemical analyzer (Beckman Coulter, AU5800, USA). The concentrations of all detected indicators were above the LOD of the instrument. The remaining serum samples were aliquoted into centrifuge tubes and stored at −80 °C.

#### 2.5.3 Liver pathological examination

After blood collection, the liver tissue of rats was rapidly dissected and isolated. The residual blood on the liver surface was rinsed with pre-cooled sterile normal saline, and the surface moisture was blotted dry with sterile filter paper. The wet weight of the liver was accurately measured, and the liver organ coefficient was calculated as follows: Liver organ coefficient (%) = (Liver wet weight / Rat body weight) × 100%. Liver tissue were immediately fixed in 4% paraformaldehyde solution at room temperature for 24 h, which were used for subsequent pathological analyses including hematoxylin-eosin (HE) staining and Masson's trichrome staining. The remaining liver tissues were rapidly aliquoted into enzyme-free cryovials, snap-frozen in liquid nitrogen, and then transferred to a −80 °C ultra-low temperature refrigerator for storage.

#### 2.5.4 Liver transcriptome sequencing

Total RNA was extracted from rat liver tissues using TRIzol reagent (Thermo Fisher Scientific, 15596018, USA). RNA purity and concentration were determined using a NanoDrop ND-1000 spectrophotometer (Wilmington, DE, USA). Subsequently, mRNA was enriched using magnetic bead-based selection, and then reverse transcribed to synthesize double-stranded cDNA. Target cDNA fragments were selected and purified using magnetic beads to construct sequencing libraries. Finally, the libraries were sequenced on the DNBSEQ-T7 platform (Majorbio Bio Technology Co., Ltd., Shanghai, China).

### 2.6 Statistical analysis

All analyses were performed using SPSS (version 22.0) or R Studio (version 4.2.1). Kolmogorov-Smirnov and Shapiro-Wilk tests were used to detect the normal distribution of heavy metals and liver function indices, and logarithmic transformations were performed on data with skewed distributions (Supplementary Tables S2). The *t*-test or Mann–Whitney *U* test was used for continuous variables, and the chi-square test was used for categorical variables.

#### 2.6.1 Multivariate linear regression model

Associations between blood heavy metal concentrations and liver function indexes were assessed using multiple linear regression. The resulting regression coefficients (β) with 95% confidence intervals (CI) represent the estimated percent change in a liver function index associated with a 1% increase in a given blood metal concentration. In addition, two models were used in the analysis: Model 1 had no adjustment variables and Model 2 was further adjusted for covariates. Furthermore, sub-group analysis was performed according to the sex of the subjects. Given the large number of association tests conducted between heavy metals and liver function indices, we performed a sensitivity analysis using the false discovery rate (FDR) approach to reduce the potential risk of false-positive findings and to assess the robustness of our results, and the results remained statistically significant after FDR correction.

#### 2.6.2 BKMR model

Given the potential non-linear and non-additive dose-response relationship between heavy metals, BKMR was used to visualize the exposure-response function and assess the combined effects of all metals on liver function (35). First, the association between single heavy metal exposure and liver function indices was assessed using the univariate exposure-response function, when all other metals were fixed at the median concentration, and the single effect of metal content was determined by estimating the change in liver function indices for each additional quartile of a single metal when other metals were fixed at the 25th, 50th, and 75th percentiles. The combined effect of the metal mixture on liver function was determined by estimating the change in the liver function indices for each 5-percentile increase/decrease in the median concentration of the metal mixture. Finally, the relative importance of different metals in liver function was determined by calculating the posterior inclusion probability (PIP), which ranges from 0 to 1. The analysis was implemented in R via the package “bkmr”. All continuous exposure variables were log-transformed and standardized to have mean 0 and standard deviation 1 to facilitate prior specification. The model was fit with 10,000 Markov chain Monte Carlo (MCMC) iterations. To assess model convergence, we computed the Geweke diagnostic for key parameters, and all Geweke z-scores were within ±1.96, indicating satisfactory convergence.

#### 2.6.3 WQS model

All blood metal concentrations were categorized into quartiles based on the distribution in the study population, with quartile 1 representing the lowest exposure level. The WQS index was constructed to estimate the combined effects of the metal mixtures on liver function. The weight that the model assigns to each metal allows the identification of important components in the effects of metal mixtures on liver function (18). Notably, WQS regression limits the effects of metal exposure on liver function in the same direction because it only tests for mixed effects that are positively or negatively correlated with a given outcome. To address potential instability due to random splitting, we employed repeated holdout validation with 100 bootstrap iterations. In each iteration, the data were randomly split into training (40%) and validation (60%) sets, weights were estimated in the training set, and the WQS index was tested in the validation set. The analysis was implemented in R via the package “gWQS”.

#### 2.6.4 Qgcomp model

Qgcomp combines WQS with g-computation to remove the restriction on directional homogeneity (19). All exposure variables were scaled to quartiles based on the empirical distribution. The model can estimate the change in liver function indices for a synchronous one-quartile increase in all heavy metals and assign a positive or negative weighted index to each heavy metal to assess the combined effect of the metal mixture. The analysis was implemented in R via the package “qgcomp”. In summary, the integrated use of these models allows for a more comprehensive and nuanced understanding of the metal mixture's effect, making our conclusions far more reliable than if we had relied on any single methodology.

## 3 Results

### 3.1 Participant characteristics

Table 1 presents the demographic characteristics of the 451 participants enrolled in the present study from the DDGXL cohort. The cohort was predominantly female, with 154 males and 297 females included. The mean age was 56.12 ± 8.09 years, and males were significantly older than females (*P* = 0.001). The mean height was 161.42 ± 7.97 cm, with males having a markedly higher mean height compared to females (*P* < 0.001). The mean weight was 61.76 ± 9.96 kg, and males were significantly heavier than females (*P* < 0.001). The mean BMI was 23.62 ± 3.44 kg/m^2^. With regard to lifestyle factors, the majority of participants were non-smokers (69.2%), non-drinkers (89.4%) and non-tea drinkers (57.0%). Gender-stratified analysis revealed that the proportions of smoking, alcohol drinking and tea drinking were significantly higher in males than in females (all *P* < 0.01).

**Table 1:** General characteristics of the study population.

Variable	All participants (*n* = 451)	Gender		*P* Value
		Male (*n* = 154)	Female (*n* = 297)	
Age (year)	56.12 (8.09)	57.91 (7.13)	55.20 (8.41)	0.001^**^
Height (cm)	161.42 (7.97)	168.93 (5.90)	157.53 (5.84)	< 0.001^**^
Weight (kg)	61.76 (9.96)	66.35 (10.03)	59.38 (9.07)	< 0.001^**^
BMI (kg/m^2^)	23.62 (3.44)	23.21 (3.00)	23.84 (3.63)	0.065
Smoking				
Yes	139 (30.8%)	77 (50.0%)	62 (20.9%)	< 0.001^**^
No	312 (69.2%)	77 (50.0%)	235 (79.1%)	
Alcohol consumption				
Yes	48 (10.6%)	25 (16.2%)	23 (7.7%)	0.009^**^
No	403 (89.4%)	129 (83.8%)	274 (92.3%)	
Tea drinking				
Yes	194 (43.0%)	84 (54.5%)	110 (37.0%)	< 0.001^**^
No	257 (57.0%)	70 (45.5%)	187 (63.0%)	
BAl (μg/L)	185.12 (24.34, 474.84)	163.30 (13.32, 445.13)	194.81 (26.39, 494.76)	0.500
BCr (μg/L)	122.05 (69.44, 137.80)	121.48 (72.20, 137.45)	122.40 (69.29, 137.90)	0.789
BCu (μg/L)	826.84 (722.22, 929.50)	803.19 (696.76, 916.82)	838.58 (726.30, 938.07)	0.065
BZn (μg/L)	5,549.23 (4,719.60, 6,231.11)	5,589.31 (4,853.06, 6,427.90)	5,543.82 (4,560.27, 6,152.05)	0.080
BAs (μg/L)	8.50 (3.69, 10.81)	8.68 (5.60, 11.15)	8.30 (3.07, 10.70)	0.092
BCd (μg/L)	0.67 (0.08, 4.01)	1.21 (0.08, 5.02)	0.42 (0.08, 3.17)	0.009^**^
BPb (μg/L)	21.62 (11.67, 36.23)	23.62 (12.66, 39.09)	20.90 (11.07, 34.74)	0.077
ALT (U/L)	21.10 (15.50, 30.20)	23.25 (16.40, 31.95)	20.80 (16.85, 29.00)	0.175
AST (U/L)	23.80 (20.20, 28.90)	23.30 (19.98, 28.25)	24.30 (20.35, 29.05)	0.276
TBil (μmol/L)	17.00 (13.80, 21.70)	18.45 (14.88, 22.90)	16.30 (13.33, 20.70)	0.002^**^
DBil (μmol/L)	6.10 (4.80, 7.20)	6.50 (5.20, 7.73)	5.80 (4.70, 6.85)	0.001^**^
IBil (μmol/L)	11.20 (8.70, 14.60)	12.00 (9.58, 15.83)	10.80 (8.45, 14.05)	0.011^*^
ALP (U/L)	90.00 (76.00, 108.00)	90.00 (78.00, 105.25)	91.00 (74.00, 108.50)	0.671
GGT (U/L)	17.00 (13.00, 27.00)	21.00 (15.75, 31.00)	16.00 (12.00, 24.00)	< 0.001^**^
CHE (KU/L)	8.85 (7.80, 10.02)	8.49 (7.50, 9.54)	9.07 (7.95, 10.18)	0.004^**^
TBA (μmol/L)	1.90 (1.40, 2.90)	1.90 (1.50, 3.20)	1.90 (1.30, 2.80)	0.175

BMI, body mass index; BAl, aluminum in blood; BCr, chromium in blood; BCu, cuprum in blood; BZn; zinc in blood; BAs, arsenic in blood; BCd, cadmium in blood; BPb, lead in blood; ALT, alanine aminotransferase; AST, aspartate aminotransferase; TBil, total bilirubin; DBil, direct bilirubin; IBil, indirect bilirubin; ALP, alkaline phosphatase; GGT, gamma glutamyl transpeptidase; CHE, cholinesterase; TBA, total bile acid.

Data were presented as *n* (%) for categorical data, mean (standard deviation) for normal distribution data, and median (interquartile range) for abnormal distribution data.

Student's *t*-test or Mann–Whitney *U* tests were used for comparison of the continuous variables according to the data distribution, and Chi-square test for the categorical variables.

^**^P < 0.01; ^*^P < 0.05.

The distribution of blood heavy metal concentrations is shown in Table 1. The median concentrations for the seven metals were 185.12 μg/L for BAl, 122.05 μg/L for BCr, 826.84 μg/L for BCu, 5549.23 μg/L for BZn, 8.50 μg/L for BAs, 0.67 μg/L for BCd, and 21.62 μg/L for BPb. Spearman's correlation analysis revealed coefficients ranging from −0.15 to 0.59 among the blood toxic metals (Supplementary Figure S2). The strongest positive correlation was observed between BCd and BPb (*r* = 0.59), while the strongest negative correlation was found between BCr and BAl (*r* = −0.15).

In this study, the median values of liver function indices, including ALT, AST, TBil, DBil, IBil, ALP, GGT, CHE, and TBA, were 21.10 U/L, 23.80 U/L, 17.00 μmol/L, 6.10 μmol/L, 11.20 μmol/L, 90.00 U/L, 17.00 U/L, 8.85 KU/L, and 1.90 μmol/L, respectively (Table 1). Spearman's correlation analysis showed coefficients ranging from −0.16 to 0.96 among the liver function indices (Supplementary Figure S3). A very strong positive correlation was found between TBil and IBil (*r* = 0.96), while the strongest negative correlation was between DBil and CHE (*r* = −0.16).

Subgroup analysis by sex indicated that male participants had significantly higher median concentrations of BCd, TBil, DBil, IBil, and GGT, but a significantly lower median serum CHE concentration compared to females (all *P* < 0.05). However, no statistically significant differences were found between sexes for the remaining blood metals or other liver function indices.

### 3.2 Associations between single metal exposure and liver function indices

**Figure 1:**
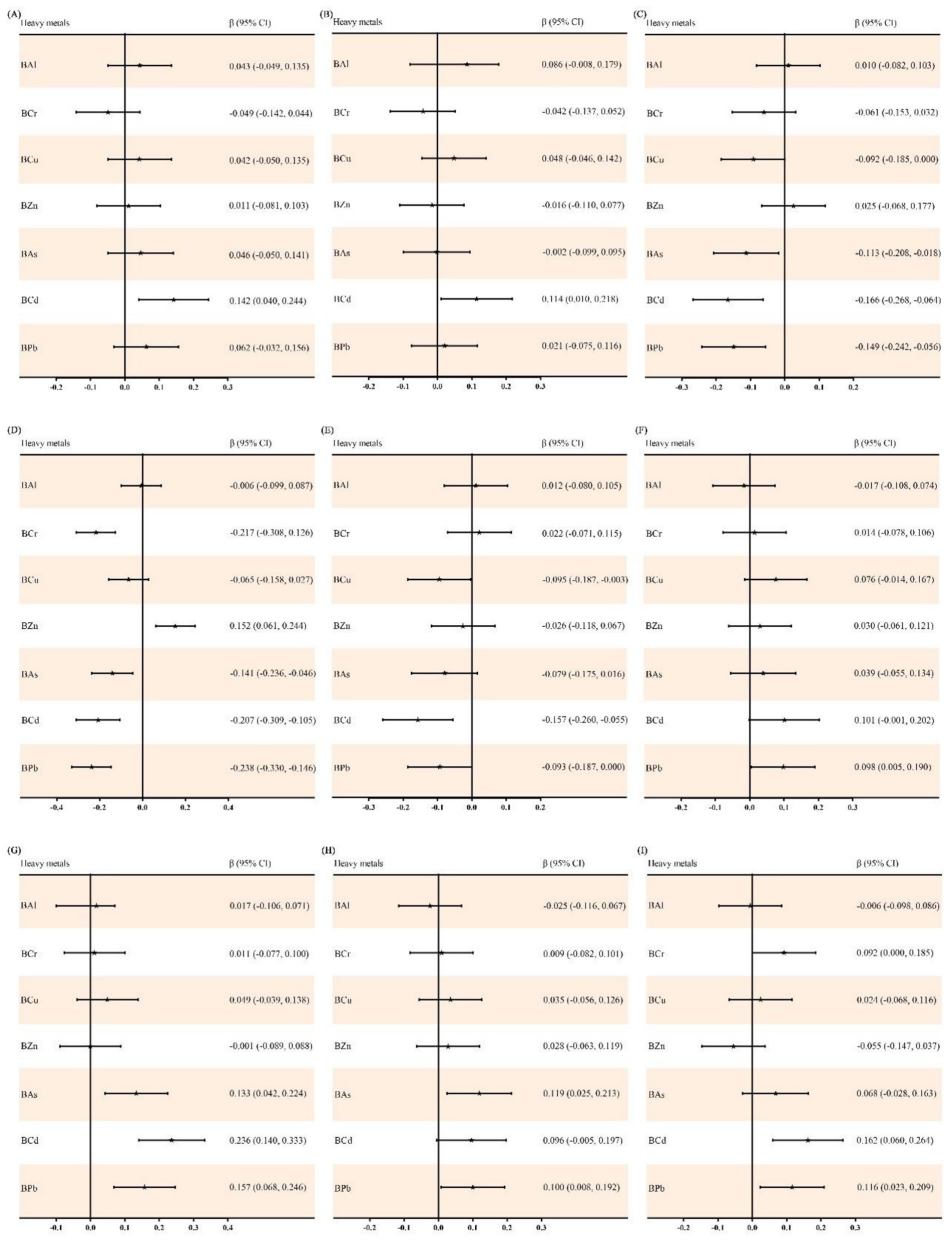
Associations between single blood heavy metal exposure and liver function indices using multiple linear regression. **(A)** ALT; **(B)** AST; **(C)** TBil; **(D)** DBil; **(E)** IBil; **(F)** ALP; **(G)** GGT; **(H)** CHE; and **(I)** TBA. Heavy metals in blood and liver function indices were log transformed. The model was adjusted for age, sex, BMI, smoking status, alcohol consumption and tea drinking. BAl, aluminum in blood; BCr, chromium in blood; BCu, cuprum in blood; BZn; zinc in blood; BAs, arsenic in blood; BCd, cadmium in blood; BPb, lead in blood; ALT, alanine aminotransferase; AST, aspartate aminotransferase; TBil, total bilirubin; DBil, direct bilirubin; IBil, indirect bilirubin; ALP, alkaline phosphatase; GGT, gamma glutamyl transpeptidase; CHE, cholinesterase; TBA, total bile acid; β: regression coefficient; CI: confidence interval.

Spearman's correlation analysis results are presented in Supplementary Table S3 and Supplementary Figure S4, which revealed weak correlations between blood heavy metal concentrations and liver function indices, with the Spearman correlation coefficients ranging from −0.21 (for BCr and DBil) to 0.22 (for BCd and GGT). Subsequently, multiple linear regression analysis was performed to evaluate the independent effects of single blood metal exposure on liver function indices. After adjusting for potential confounding factors including age, sex, BMI, smoking status, alcohol consumption and tea drinking, significant associations were observed as follows: BCd was positively correlated with GGT (β = 0.236), TBA (β = 0.162), ALT (β = 0.142) and AST (β = 0.114), while negatively correlated with DBil (β = −0.207), TBil (β = −0.166) and IBil (β = −0.157); BPb was positively correlated with GGT (β = 0.157), TBA (β = 0.116), CHE (β = 0.100) and ALP (β = 0.098), and negatively correlated with DBil (β = −0.238), TBil (β = −0.149) and IBil (β = −0.093); BAs was positively correlated with GGT (β = 0.133) and CHE (β = 0.119), whereas negatively correlated with DBil (β = −0.141) and TBil (β = −0.113); BCr was positively correlated with TBA (β = 0.092) and negatively correlated with DBil (β = −0.217); BCu was negatively correlated with IBil (β = −0.095) and TBil (β = −0.092); BZn was only positively correlated with DBil (β = 0.152). All the aforementioned associations reached statistical significance (all *P* < 0.05) (Supplementary Table S4 and Figure 1). Subgroup analysis stratified by sex confirmed consistent association trends between individual blood metals and liver function indices across all subgroups (Supplementary Table S5).

### 3.3 Associations between mixed metal exposure and liver function indices

#### 3.3.1 BKMR model

**Figure 2:**
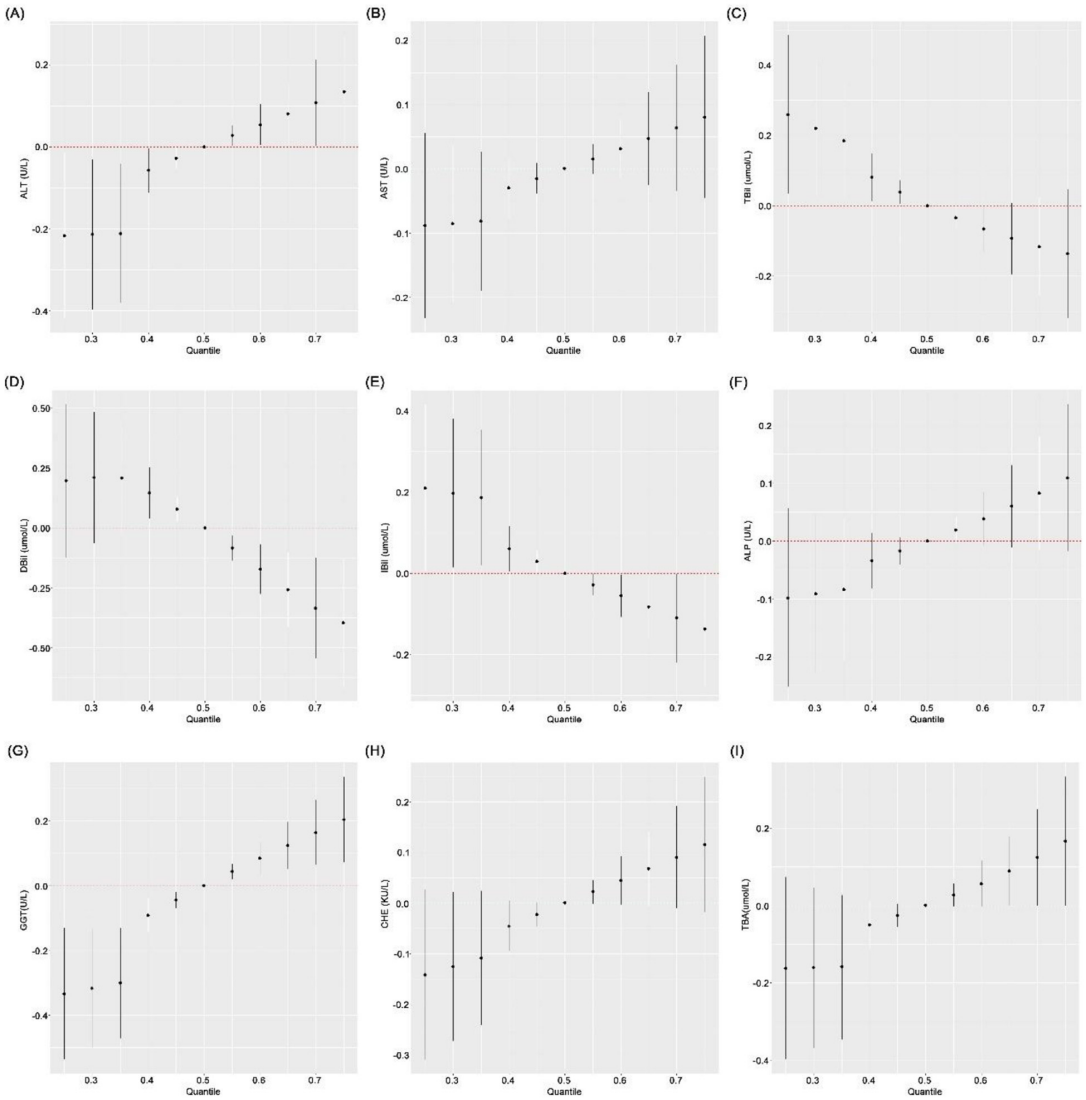
Overall effect of blood heavy metal mixture on liver function indices by BKMR model. The figure shows the estimated change (with 95% confidence intervals) in each liver function index when all seven blood heavy metals are simultaneously increased from their median concentrations. A positive estimate indicates that increasing the metal mixture is associated with higher levels of the liver function index, while a negative estimate indicates an inverse association. **(A)** ALT; **(B)** AST; **(C)** TBil; **(D)** DBil; **(E)** IBil; **(F)** ALP; **(G)** GGT; **(H)** CHE; and **(I)** TBA. Heavy metals in blood and liver function indices were log transformed. The model was adjusted for age, sex, BMI, smoking status, alcohol consumption and tea drinking. BAl, aluminum in blood; BCr, chromium in blood; BCu, cuprum in blood; BZn; zinc in blood; BAs, arsenic in blood; BCd, cadmium in blood; BPb, lead in blood; ALT, alanine aminotransferase; AST, aspartate aminotransferase; TBil, total bilirubin; DBil, direct bilirubin; IBil, indirect bilirubin; ALP, alkaline phosphatase; GGT, gamma glutamyl transpeptidase; CHE, cholinesterase; TBA, total bile acid; β: regression coefficient; CI: confidence interval.

**Figure 3:**
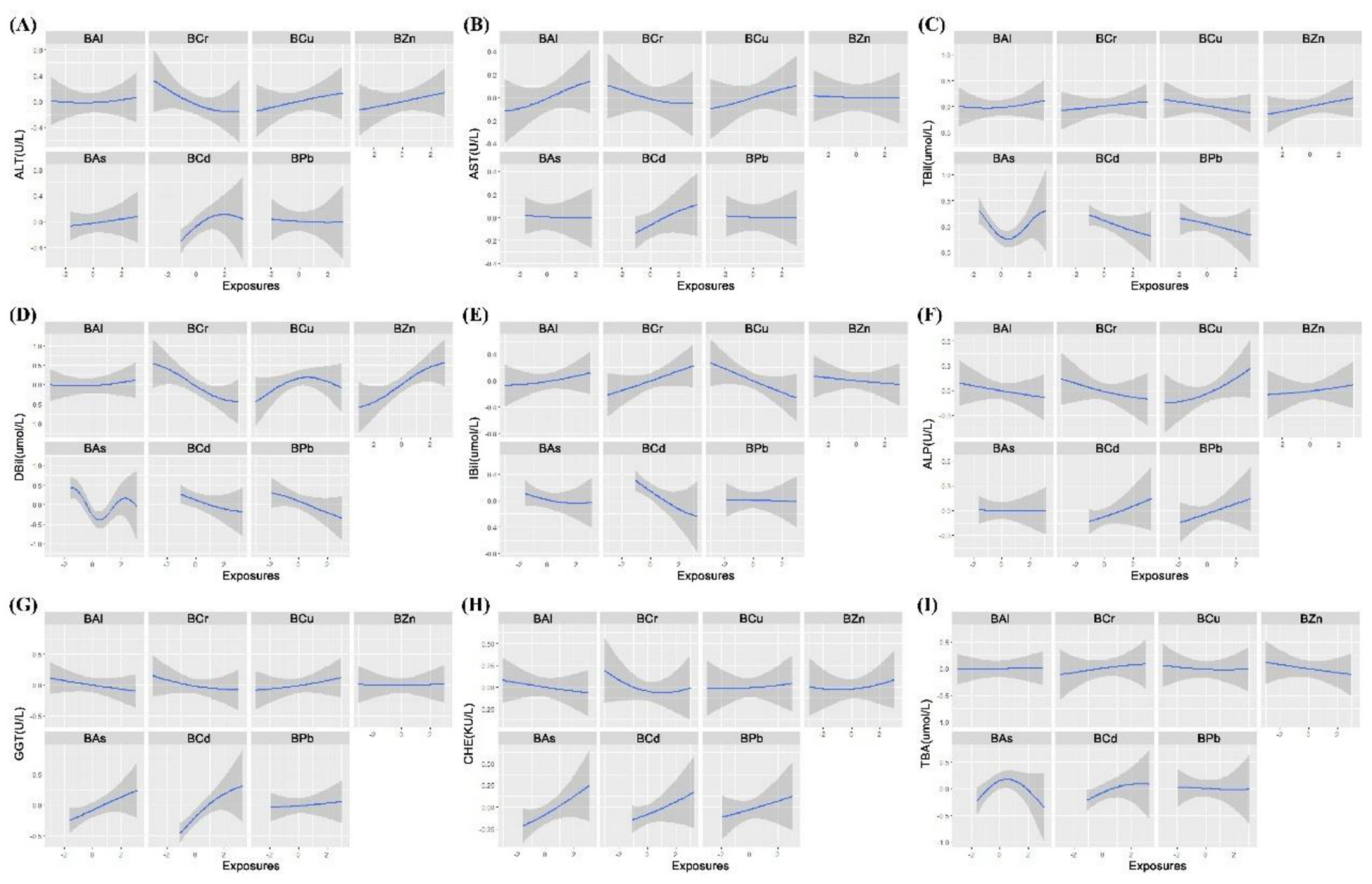
Univariate exposure–response functions for individual blood heavy metals by BKMR model. Each panel displays the estimated relationship (with 95% confidence intervals) between a single blood heavy metal and a liver function index, with all other metals fixed at their median concentrations. A curve that deviates from the horizontal line at zero indicates an association; the direction of the deviation (positive or negative) indicates the direction of the association, and the shape of the curve reveals potential non-linearity. **(A)** ALT; **(B)** AST; **(C)** TBil; **(D)** DBil; **(E)** IBil; **(F)** ALP; **(G)** GGT; **(H)** CHE; and **(I)** TBA. Heavy metals in blood and liver function indices were log transformed. The model was adjusted for age, sex, BMI, smoking status, alcohol consumption and tea drinking. BAl, aluminum in blood; BCr, chromium in blood; BCu, cuprum in blood; BZn; zinc in blood; BAs, arsenic in blood; BCd, cadmium in blood; BPb, lead in blood; ALT, alanine aminotransferase; AST, aspartate aminotransferase; TBil, total bilirubin; DBil, direct bilirubin; IBil, indirect bilirubin; ALP, alkaline phosphatase; GGT, gamma glutamyl transpeptidase; CHE, cholinesterase; TBA, total bile acid.

**Figure 4:**
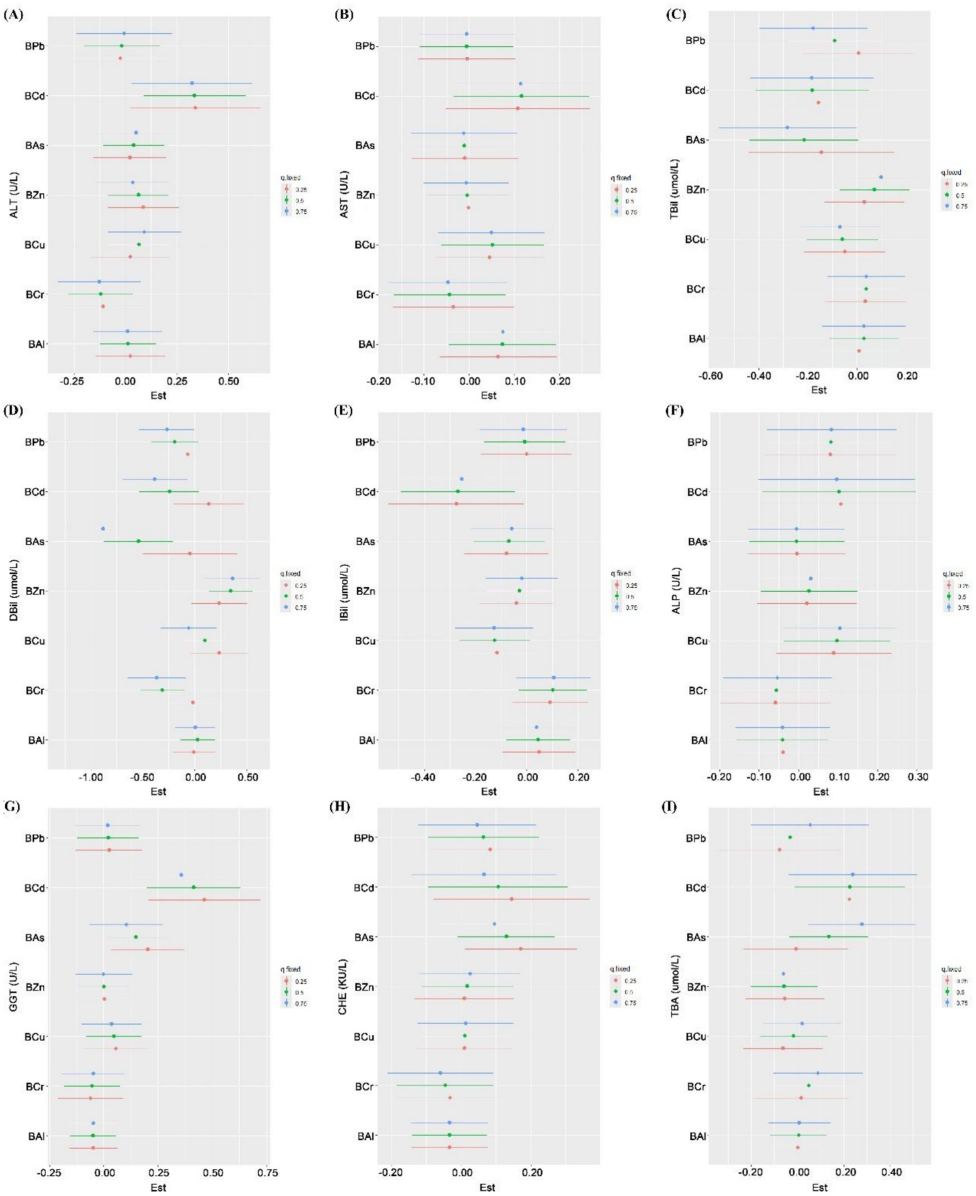
Single-exposure effects of individual blood heavy metals at different mixture percentiles by BKMR model. This figure shows the estimated change (with 95% confidence intervals) in each liver function index associated with a one-quartile increase in a single metal, when the concentrations of the remaining six metals are fixed at the 25th (red), 50th (green), or 75th (blue) percentile. **(A)** ALT; **(B)** AST; **(C)** TBil; **(D)** DBil; **(E)** IBil; **(F)** ALP; **(G)** GGT; **(H)** CHE; and **(I)** TBA. Heavy metals in blood and liver function indices were log transformed. The model was adjusted for age, sex, BMI, smoking status, alcohol consumption and tea drinking. BAl, aluminum in blood; BCr, chromium in blood; BCu, cuprum in blood; BZn; zinc in blood; BAs, arsenic in blood; BCd, cadmium in blood; BPb, lead in blood; ALT, alanine aminotransferase; AST, aspartate aminotransferase; TBil, total bilirubin; DBil, direct bilirubin; IBil, indirect bilirubin; ALP, alkaline phosphatase; GGT, gamma glutamyl transpeptidase; CHE, cholinesterase; TBA, total bile acid.

**Figure 5:**
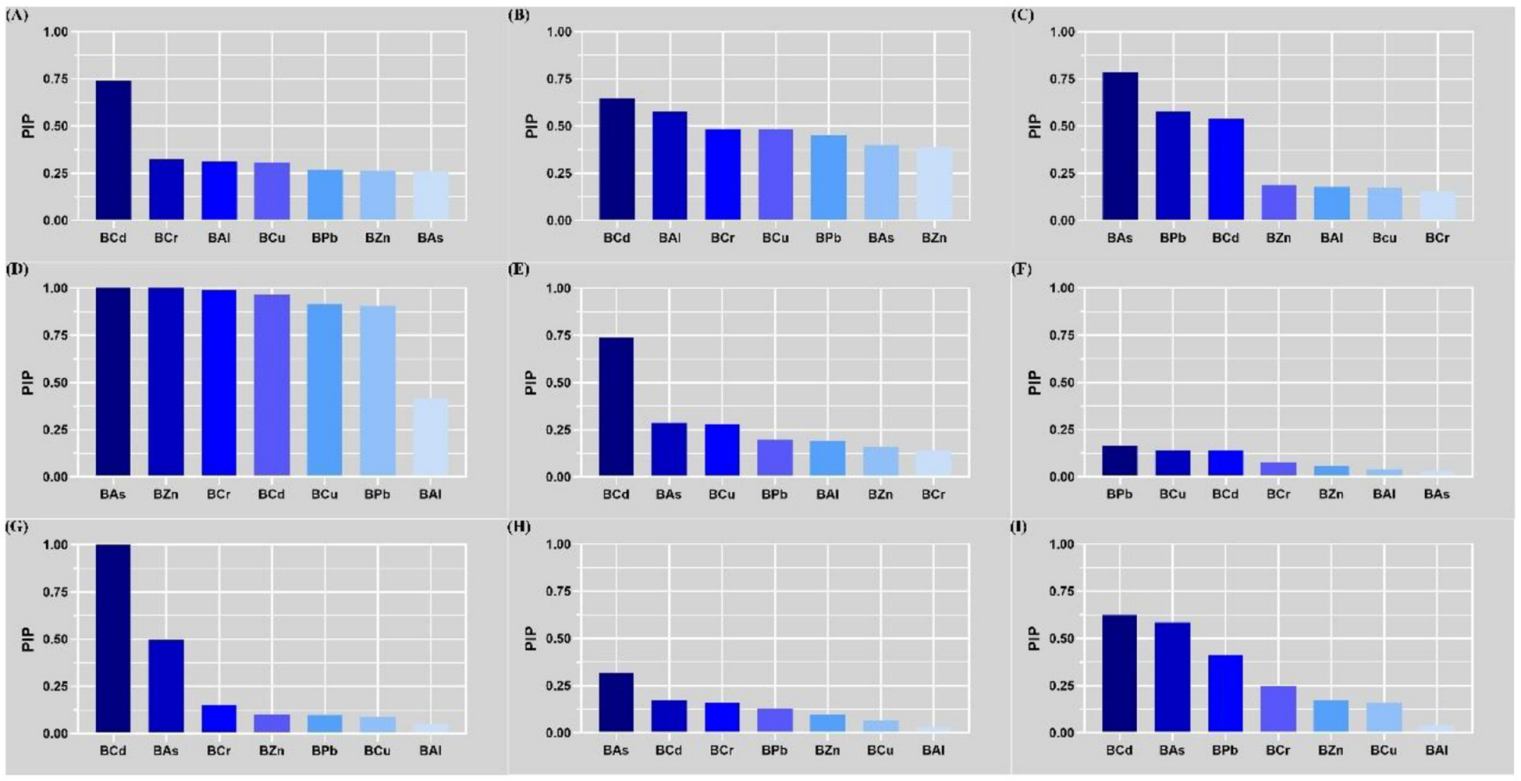
PIP values of each blood heavy metal in the BKMR model. PIP values range from 0 to 1 and indicate the relative importance of each metal in the mixture for explaining variation in each liver function index. Higher PIP values (closer to 1) suggest a stronger contribution of that metal to the observed association. **(A)** ALT; **(B)** AST; **(C)** TBil; **(D)** DBil; **(E)** IBil; **(F)** ALP; **(G)** GGT; **(H)** CHE; and **(I)** TBA. Heavy metals in blood and liver function indices were log transformed. The model was adjusted for age, sex, BMI, smoking status, alcohol consumption and tea drinking. BAl, aluminum in blood; BCr, chromium in blood; BCu, cuprum in blood; BZn; zinc in blood; BAs, arsenic in blood; BCd, cadmium in blood; BPb, lead in blood; ALT, alanine aminotransferase; AST, aspartate aminotransferase; TBil, total bilirubin; DBil, direct bilirubin; IBil, indirect bilirubin; ALP, alkaline phosphatase; GGT, gamma glutamyl transpeptidase; CHE, cholinesterase; TBA, total bile acid; PIP, posterior inclusion probability.

Multiple mixture models were applied to estimate the overall effects of blood heavy metal mixtures on liver function. After adjusting for confounding variables in the BKMR model, cumulative effect curves revealed positive associations between exposure to blood heavy metal mixtures and serum ALT, AST, ALP, GGT, CHE and TBA, as well as negative associations with TBil, DBil and IBil. Specifically, compared with the median concentration of metal mixtures, serum levels of ALT, AST, ALP, GGT, CHE and TBA exhibited a gradual increasing trend with the elevation of blood metal mixture concentrations, whereas serum TBil, DBil and IBil showed a decreasing trend (Figure 2). The exposure-response relationships between individual metals and liver function indices were further evaluated, with the corresponding curves presented in Figure 3. When concentrations of the other metals were fixed at their median values, BCd was positively correlated with ALT, AST, ALP, GGT, CHE and TBA, and negatively correlated with TBil, DBil and IBil, with these associations being more pronounced at relatively low BCd concentrations. BPb was positively associated with ALP and CHE, and negatively associated with TBil and DBil. BAs showed positive correlations with GGT, CHE and TBA, and negative correlations with TBil and DBil. BZn was positively correlated with DBil. These findings were consistent with the results of multiple linear regression analysis. After fixing the concentrations of the seven metals at the 25th, 50th and 75th percentiles, respectively, the associations between a one-unit increase in the concentration of each individual metal and liver function indices were illustrated in Figure 4. The results showed that BCd was positively correlated with ALT and GGT, and negatively correlated with IBil, when the concentrations of the remaining metals increased from the 25th to the 75th percentile. Additionally, BAs was positively associated with TBA and negatively associated with TBil and DBil when the remaining metals were fixed at the 50th and 75th percentiles (all *P* < 0.05). Finally, the PIP values were calculated to quantify the contribution of each individual metal in the mixture to liver function indices. The results demonstrated that BCd had the highest PIP values and thus was the top contributor to ALT, AST, IBil, GGT and TBA; BAs was the primary contributor to TBil, DBil and CHE; and BPb was the major contributor to ALP (Supplementary Table S6 and Figure 5).

#### 3.3.2 WQS model

**Figure 6:**
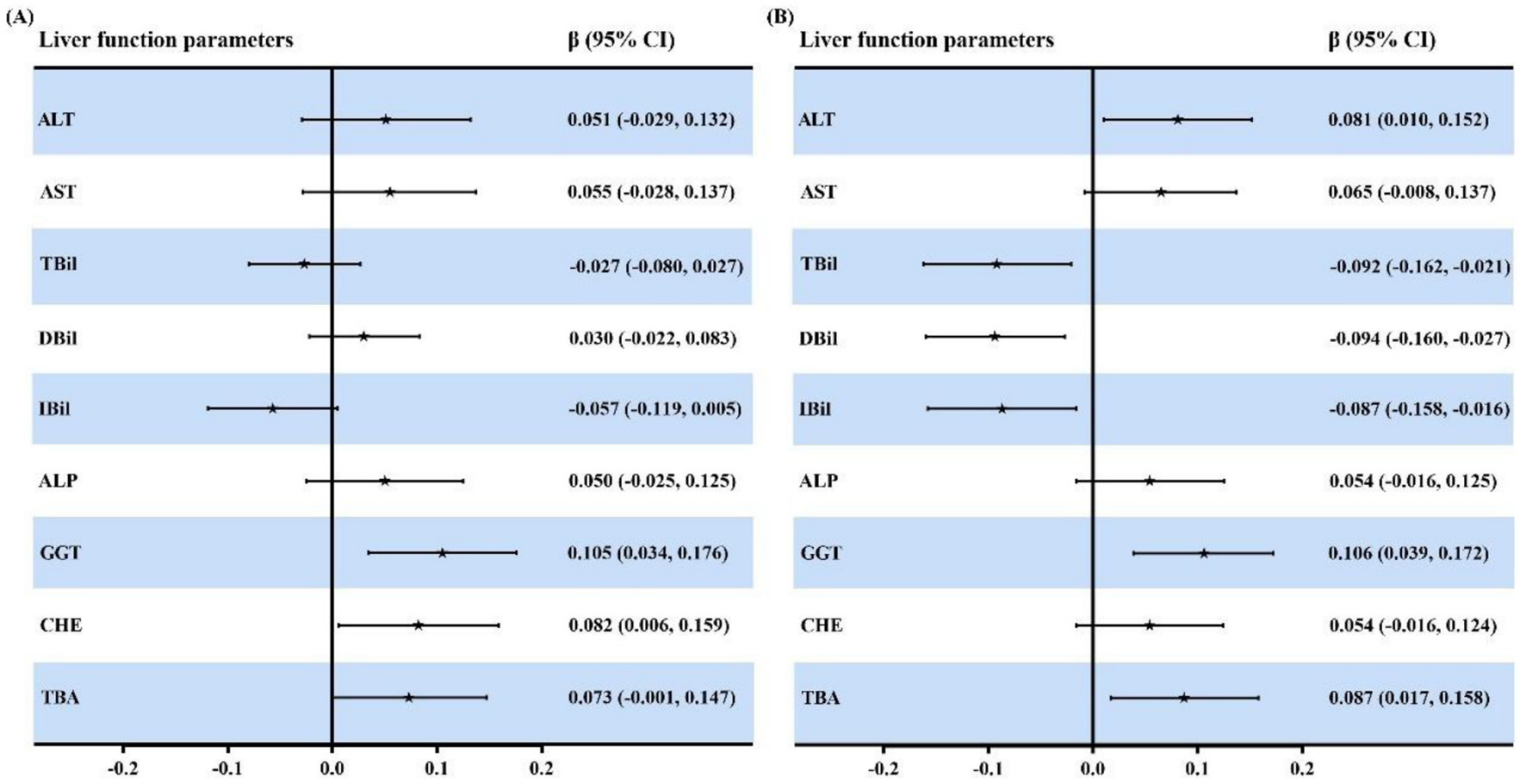
Overall association between blood heavy metal mixture and liver function indices by WQS and qgcomp models. **(A)** WQS regression results: The plot shows the estimated change (with 95% confidence intervals) in each liver function index associated with a one-quartile increase in the WQS index. **(B)** qgcomp results: The plot shows the estimated change (with 95% confidence intervals) in each liver function index associated with a simultaneous one-quartile increase in all seven metals, allowing for both positive and negative contributions. Heavy metals in blood and liver function indices were log transformed. The model was adjusted for age, sex, BMI, smoking status, alcohol consumption and tea drinking. BAl, aluminum in blood; BCr, chromium in blood; BCu, cuprum in blood; BZn; zinc in blood; BAs, arsenic in blood; BCd, cadmium in blood; BPb, lead in blood; β: regression coefficient; CI: confidence interval.

**Figure 7:**
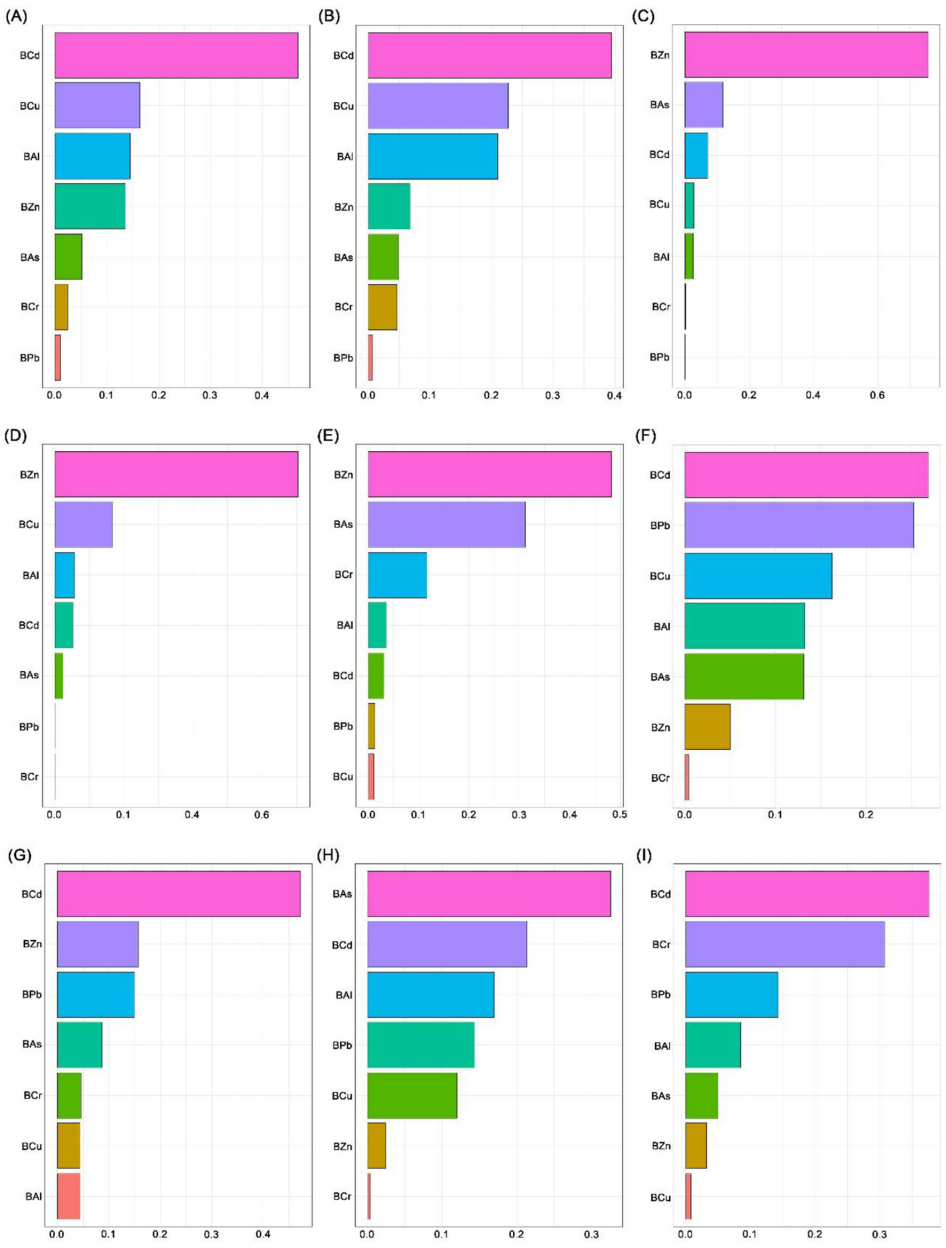
Weight values of blood heavy metal for liver function indices by WQS models. The weights (ranging from 0 to 1) indicate the relative contribution of each metal to the overall mixture effect estimated by WQS regression. Higher weights suggest that the metal is a more important contributor to the association. All weights for a given outcome sum to 1. **(A)** ALT; **(B)** AST; **(C)** TBil; **(D)** DBil; **(E)** IBil; **(F)** ALP; **(G)** GGT; **(H)** CHE; and **(I)** TBA. Heavy metals in blood and liver function indices were log transformed. The model was adjusted for age, sex, BMI, smoking status, alcohol consumption and tea drinking. BAl, aluminum in blood; BCr, chromium in blood; BCu, cuprum in blood; BZn; zinc in blood; BAs, arsenic in blood; BCd, cadmium in blood; BPb, lead in blood; ALT, alanine aminotransferase; AST, aspartate aminotransferase; TBil, total bilirubin; DBil, direct bilirubin; IBil, indirect bilirubin; ALP, alkaline phosphatase; GGT, gamma glutamyl transpeptidase; CHE, cholinesterase; TBA, total bile acid.

The WQS model was used to quantify the combined effect of blood metal mixtures on liver function indices by constructing a weighted index. After adjusting for confounding variables, the results showed that the WQS index of blood heavy metal mixtures was significantly positively correlated with GGT (β = 0.105) and CHE (β = 0.082) (all *P* < 0.05) (Supplementary Table S7 and Figure 6). By assigning weights to all heavy metals, the relative contribution of each individual metal to the observed effects on liver function indices was further identified, BCd was the primary contributor to ALT (46.79%), AST (39.37%), ALP (26.83%), GGT (47.14%) and TBA (37.54%); BZn was the dominant contributor to TBil (75.57%), DBil (70.31%) and IBil (48.14%); and BAs was the top contributor to CHE (32.53%) (Supplementary Table S8 and Figure 7).

#### 3.3.3 Qgcomp model

**Figure 8:**
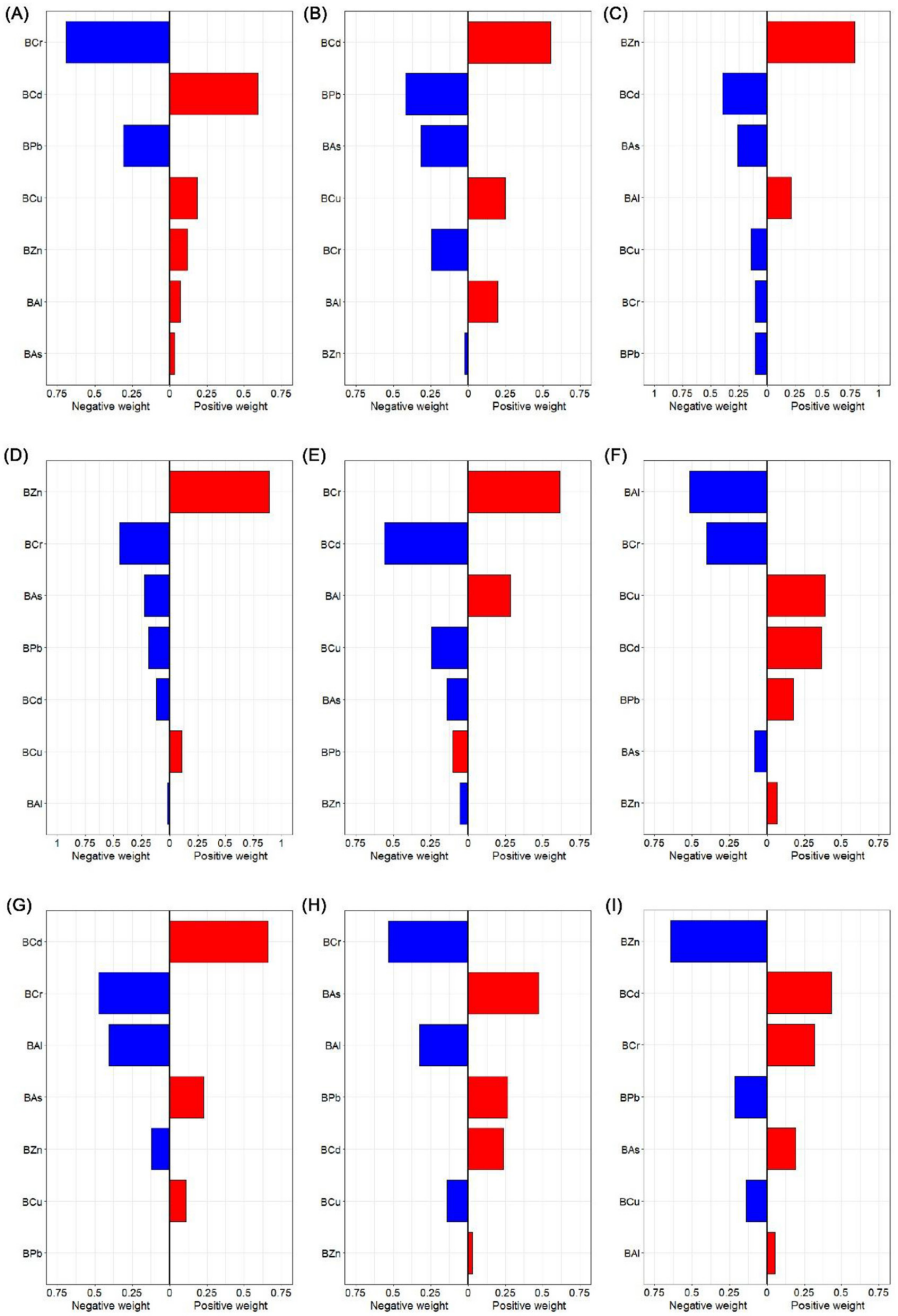
Weight values of blood heavy metals for liver function indices by Qgcomp models. The weights can be positive (indicating a positive contribution to the overall mixture effect) or negative (indicating an inverse contribution). Weights for positive and negative contributions are shown separately, and within each direction, the weights sum to 1. **(A)** ALT; **(B)** AST; **(C)** TBil; **(D)** DBil; **(E)** IBil; **(F)** ALP; **(G)** GGT; **(H)** CHE; and **(I)** TBA. Heavy metals in blood and liver function indices were log transformed. The model was adjusted for age, sex, BMI, smoking status, alcohol consumption and tea drinking. BAl, aluminum in blood; BCr, chromium in blood; BCu, cuprum in blood; BZn; zinc in blood; BAs, arsenic in blood; BCd, cadmium in blood; BPb, lead in blood; ALT, alanine aminotransferase; AST, aspartate aminotransferase; TBil, total bilirubin; DBil, direct bilirubin; IBil, indirect bilirubin; ALP, alkaline phosphatase; GGT, gamma glutamyl transpeptidase; CHE, cholinesterase; TBA, total bile acid.

The Qgcomp model was employed to assess the combined effect of blood heavy metal mixtures on liver function indices. After adjusting for confounding variables, the results demonstrated that each one-quartile increase in the blood metal mixture was significantly positively associated with ALT (β = 0.081), GGT (β = 0.106) and TBA (β = 0.087), while significantly negatively associated with TBil (β = −0.092), DBil (β = −0.094) and IBil (β = −0.087) (all *P* < 0.05) (Supplementary Table S9 and Figure 6). By assigning weights to each metal, the relative contribution of individual metals to the observed associations with liver function indices was quantified. The results showed that BCd had the highest weights in the positive associations with ALT (weight = 0.593), AST (weight = 0.555), GGT (weight = 0.661) and TBA (weight = 0.434), as well as in the negative associations with TBil (weight = −0.395) and IBil (weight = −0.558). BCr exhibited the highest weight in the positive association with IBil (weight = 0.617) and the highest weights in the negative associations with ALT (weight = −0.693), DBil (weight = −0.449), GGT (weight = −0.473) and CHE (weight = −0.534). BZn had the highest weights in the positive associations with TBil (weight = 0.784) and DBil (weight = 0.889), and the highest weight in the negative association with TBA (weight = −0.645). BPb showed the highest weight in the negative association with AST (weight = −0.420). BCu had the highest weight in the positive association with ALP (weight = 0.390). BAs exhibited the highest weight in the positive association with CHE (weight = 0.470). BAl had the highest weight in the negative association with ALP (weight = −0.517) (Supplementary Table S10 and Figure 8).

### 3.4 *In vivo* experimental validation of Cd-induced liver injury

**Figure 9:**
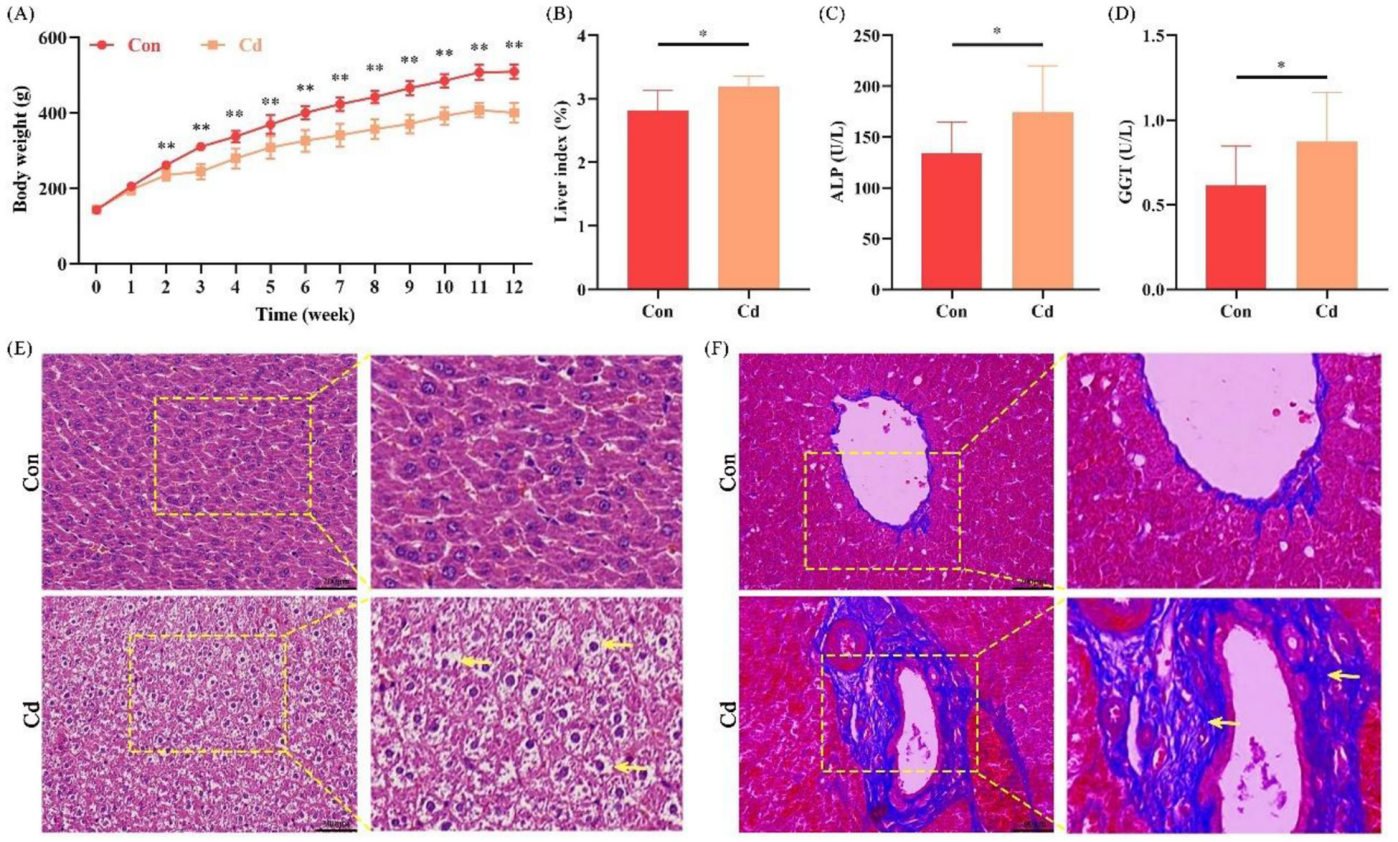
Cd exposure induces liver injury in rats (*n* = 7). **(A)** Effect of Cd exposure on weight of rats; **(B)** Effect of Cd exposure on liver coefficient in rats; **(C)** Serum levels of ALP; **(D)** Serum levels of GGT; **(E)** HE staining; **(F)** Masson's trichrome staining. *P < 0.05; **P < 0.01. Cd, cadmium; ALP, alkaline phosphatase; GGT, gamma glutamyl transpeptidase; HE, Hematoxylin and Eosin.

Based on the aforementioned epidemiological findings in the human cohort, Cd was identified as the predominant metal associated with liver function biomarkers of liver injury induced by combined heavy metal exposure. Therefore, a sub-chronic Cd exposure rat model was established for *in vivo* validation. The results showed that the body weight gain rate of rats in the Cd treated group was significantly lower than that in the control group starting from the second week of exposure (*P* < 0.01) (Figure 9A). By weighing the rat liver, it was found that the liver organ coefficient of rats in the Cd treated group was significantly decreased compared with the control group (*P* < 0.05) (Figure 9B). Further detection of serum liver function indices revealed that the serum levels of ALP and GGT in the Cd treated group were significantly higher than those in the control group (both *P* < 0.05) (Figures 9C, D). The results of liver histopathological analysis were as follows: HE staining showed that the liver tissue structure of rats in the control group was intact, with regular arrangement of hepatic lobules, normal hepatocyte morphology, clear cell nuclei and uniform nucleocytoplasmic staining. In contrast, the liver tissue architecture was disorganized in the Cd treated group, and hepatocytes exhibited significant cytoplasmic vacuolar degeneration (Figure 9E). Masson's trichrome staining was used to assess hepatic collagen fiber deposition. As shown in Figure 9F, the area of collagen fiber deposition (stained blue) in the liver tissue of the Cd treated group was significantly increased compared with the control group, and the deposited collagen fibers were mainly concentrated around the portal areas. Collectively, these *in vivo* experimental results confirm that long-term low-dose Cd exposure can induce liver injury in rats.

### 3.5 The underlying mechanism of the Cd-induced liver injury

**Figure 10:**
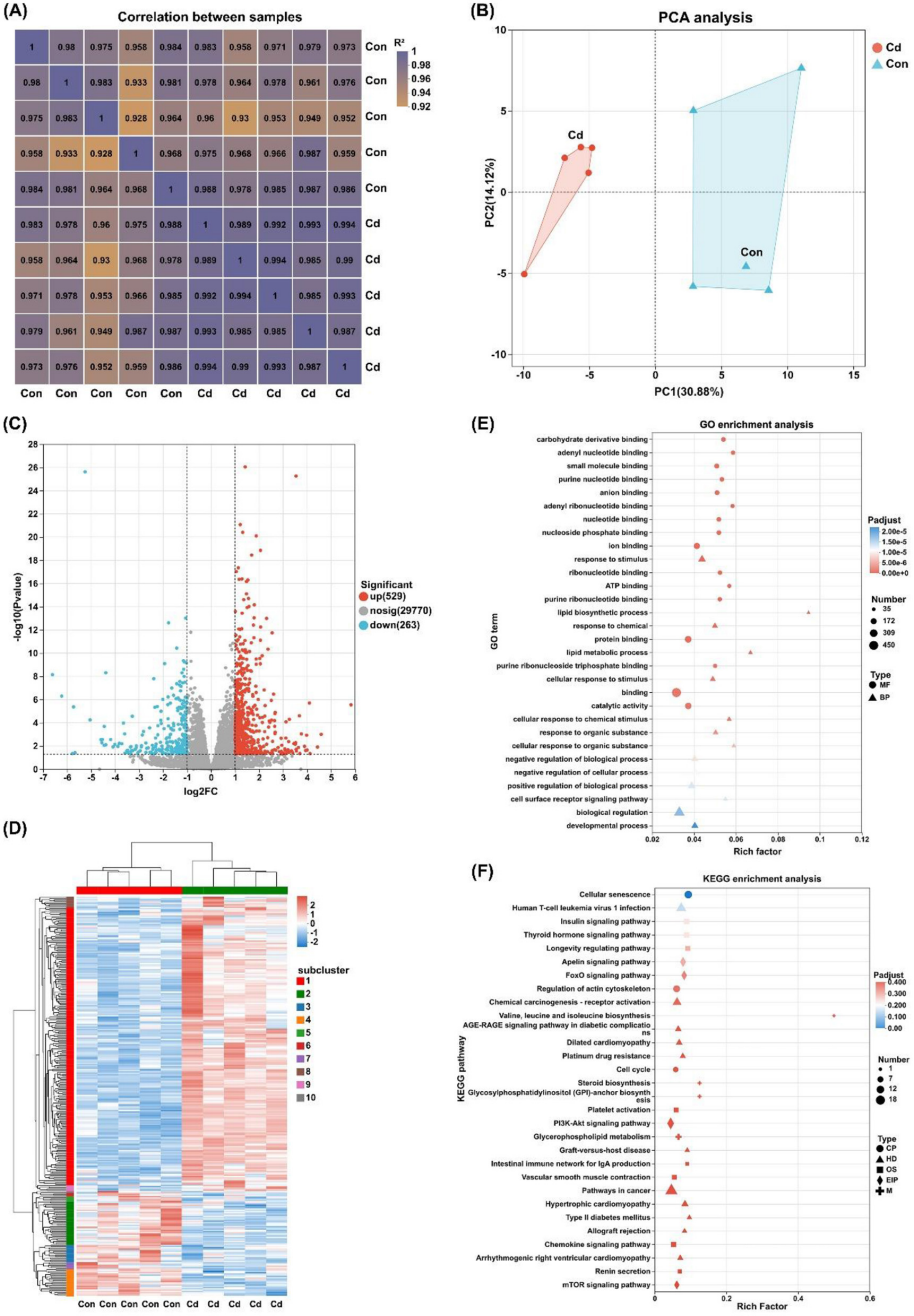
Mechanism characteristics and bioinformatics analysis of Cd exposure induces liver injury. (n = 5). **(A)** Correlation analysis heatmap; **(B)** Principal component analysis; **(C)** Volcano map of differentially expressed genes; **(D)** Heatmaps of gene expression clustering; **(E)** GO enrichment analysis; **(F)** KEGG analysis. Abbreviations: Cd, cadmium.

The correlation of gene expression levels among samples is a critical indicator to evaluate experimental reliability and the rationality of sample selection. As shown in Figure 10A, the coefficient of determination (*R*^2^) among all biological replicate samples was >0.92, indicating a strong correlation in gene expression across samples with high data reliability, which is suitable for subsequent in-depth analysis. Principal component analysis (PCA) was performed based on gene expression levels of all samples. As illustrated in Figure 10B, no overlap was observed between sample points of the control group and the Cd treated group, showing a distinct intergroup separation trend. Volcano map revealed that a total of 792 differentially expressed genes (DEGs) were identified in the Cd treated group compared with the control group (Figure 10C), including 529 upregulated genes and 263 downregulated genes. Hierarchical clustering analysis was performed on all DEGs, and a heatmap was generated (Figure 10D). Intra-group clustering showed that samples in the same group clustered into one cluster due to similar gene expression patterns. Inter-group comparison revealed that the gene expression patterns between the Cd treated group and the control group were significantly different, which were divided into distinct clustering branches. This further confirms that Cd exposure can significantly alter the mRNA expression profile of liver tissue. Gene ontology (GO) functional enrichment analysis was conducted for the DEGs. The top 30 significantly enriched pathways were mainly concentrated in the two categories of biological process (BP) and molecular function (MF) (Figure 10E). Specifically, the significantly enriched terms in the BP category included response to stimulus, lipid biosynthetic process, response to chemical, and lipid metabolic process. The significantly enriched terms in the MF category included carbohydrate derivative binding, adenyl nucleotide binding, small molecule binding, and purine nucleotide binding. Kyoto encyclopedia of genes and genomes (KEGG) pathway enrichment analysis showed that the top 30 significantly enriched pathways included Steroid biosynthesis, Glycerophospholipid metabolism, and FoxO signaling pathway (Figure 10F). Collectively, the transcriptomic analysis results further confirm that chronic low-dose Cd exposure can induce liver injury in rats, and lipid dysregulation may play a potential role.

## 4 Discussion

The present study is among the latest investigations evaluating the association between environmental heavy metal mixture exposure and liver function indices in rural residents of northwestern China. Multiple linear regression analysis revealed that BCd was significantly positively correlated with GGT, TBA, ALT, and AST, while negatively correlated with DBil, TBil, and IBil. More importantly, BKMR, WQS regression, and Qgcomp yielded consistent results that a significant positive association between blood heavy metal mixture exposure and GGT, and collectively identified Cd as the predominant metal associated with liver function biomarkers. Furthermore, *in vivo* experiments using a sub-chronic low-dose Cd exposure rat model supported the link between Cd exposure and liver injury. Transcriptome sequencing results further deciphered that lipid dysregulation may play a potential role underlying Cd-induced liver injury. Taken together, this study provides novel, multi-dimensional epidemiological and experimental evidence for clarifying the combined effects of environmental heavy metal mixture exposure on liver function in rural residents of northwestern China.

The global burden of chronic liver disease poses enormous health challenges in many countries. In 2017, 1.5 billion people worldwide suffered from chronic liver disease (5) and its prevalence increases with age, peaking in individuals aged 45 and above for men and women (4, 36). In addition, individuals aged over 40 are more sensitive to exogenous exposure to environmental heavy metals than other age groups (37). Therefore, over 40s were ideal participants for this study. Previous studies, mostly in adults older than 18 years, found that heavy metal exposure in the blood or urine led to elevated liver enzymes and showed a significant dose-response relationship, which is consistent with our findings (25, 38). Other studies pointed out that exposure to heavy metals, including Cd and Zn, increases the risk of liver injury in adolescents (12–19 years old), which is probably mediated by cholesterol (39, 40). This study focused on middle-aged and older adults, further supplementing the research results on the effects of heavy metal exposure on liver function.

The liver is an important organ responsible for the metabolism and detoxification of toxic substances and is a target organ for heavy metal accumulation (41). The accumulation of toxic metals in the liver induces hepatocyte toxicity (42), which increases the risk of chronic liver diseases such as NAFLD (43). The liver function test is a common method for liver function assessment, including ALT, AST, and bilirubin levels, and is of great value for the early diagnosis of liver diseases. In this study, several heavy metals, particularly BCd and BPb, were significantly positively correlated with ALT, AST, GGT, ALP, CHE, and TBA levels. Consistent with our results, several cross-sectional studies from China, South Korea, and the United States have shown that Cd and Pb can increase liver enzyme levels, thereby impairing liver function (25, 44, 45). In addition, complex relationships were observed between BAs, BCr, BCu, and liver function indices, suggesting that heavy metal exposure can affect liver health. However, blood heavy metal concentrations showed a negative relationship with bilirubin, which contradicts the common notion that bilirubin is elevated during liver injury. Previous studies in humans and animals have generally shown that heavy metal exposure causes elevated bilirubin levels (46, 47). Recent studies suggested that bilirubin is an antioxidant that directly removes reactive oxygen species (ROS) and inhibits oxidative damage (48). The slight decrease in the bilirubin levels may be a form of oxidative stress caused by the metal mixture. Reductions in bilirubin levels have also been observed in people exposed to other heavy metals or chemical poisons (49, 50). However, this interpretation remains speculative in the context of our study, as we did not directly measure oxidative stress biomarkers. Furthermore, the observed reductions in bilirubin levels may also be attributable to other factors, including residual confounding (e.g., nutritional status, hemolysis-related factors), statistical artifacts arising from highly correlated biomarkers, and direct alterations in bilirubin metabolic pathways. Future studies incorporating direct measurements of oxidative stress biomarkers, comprehensive dietary assessments, and repeated bilirubin measurements will help elucidate the underlying mechanisms.

It is important to emphasize that while the selected metals are individually established hepatotoxicants, the primary aim of this study was to advance the understanding of their combined effects, as environmental exposure to metal mixtures represents a more realistic and pressing public health challenge. In this context, new statistical models have emerged that have deepened research on mixtures (17). In this study, the results regarding the significant association between the blood heavy metal mixture and liver function indices were similar across the three models, indicating that they were reliable. Previous studies have also reported differences in the combined effects of mixed-metal exposure across different models. For example, Guo et al. (20) found that the effects of multiple urinary metal exposures on cardiovascular disease identified different important metals in different statistical models. A study on the association of urinary metal mixtures with NAFLD and liver fibrosis also reached consistent conclusions (51). Therefore, multiple statistical methods should be used to simultaneously evaluate the binding effects of metal mixtures at the same time, and the most appropriate interpretation of the results should be made after balancing their advantages and disadvantages. In addition, after a comprehensive analysis of the three models, we determined that BCd was the most important components of the heavy metals mixture, further suggesting a toxic effect of heavy metals on liver function. However, the relationship between metal mixtures and liver function indices in different studies may yield inconsistent results, which are related to the study population, biological samples, and metal interactions. Therefore, it is necessary to investigate the combined effects of metal mixtures on liver function.

The application of multiple mixture models in this study was deliberate and served to enhance the robustness of our findings. Notably, the three models provided complementary insights: BKMR revealed non-linear exposure-response relationships and allowed visual inspection of potential interactions; however, the possibility of more complex or subtler interactive effects cannot be ruled out and warrants investigation in larger studies (52). WQS and qgcomp provided quantitative estimates of the overall mixture effect and the relative contribution of each metal; however, WQS assumes that the direction of the metal mixture effect is uniform and is not as flexible as BKMR (53), whereas qgcomp can simultaneously observe metal interactions in both positive and negative directions (54). The observed discrepancies across models are informative rather than problematic. In the present study, while WQS imposes the assumption of unidirectional effects and identified BCd as the dominant contributor to positive associations, qgcomp relaxes this assumption and additionally revealed that BZn may contribute in the opposite direction for certain outcomes. Similarly, the dominance of BAs for bilirubin indices in BKMR and CHE in all models, but its lesser role in WQS, may reflect non-linear relationships that BKMR captures but WQS does not. Importantly, despite these model-specific differences, Cd emerged as the most consistently implicated metal across outcomes and models, particularly for enzymes indicative of hepatocellular injury (ALT, AST, GGT) and bile acids (TBA). This consistency, observed across three models with different assumptions, strengthens confidence in Cd as a key contributor to liver function alterations in this population. These findings underscore the value of using multiple complementary models in mixture research and highlight the importance of interpreting results with attention to both convergence and divergence across methods.

Although many studies have been conducted on the hepatotoxicity caused by heavy metals, oxidative stress is considered the most plausible explanation. Heavy metals lead to hepatocyte toxicity by inducing excessive production of ROS, inducing oxidative stress transcription factors, destroying antioxidant defense system, and accelerating lipid peroxidation and other pathways (9, 42). Cd interferes with the balance of the liver redox state and induces the activation of stellate cells, leading to liver fibrosis (55). Cr (VI) causes liver genotoxicity by inducing oxidative stress and disrupts liver histopathological and ultrastructural features (56). In addition, both *in vivo* and *in vitro* experiments have reported an important role of oxidative stress in liver injury caused by Pb, As, and Mn (57–60). Furthermore, emerging evidence highlights the critical role of systemic inflammation as a mediating pathway linking metal exposure to liver injury. A large study based on the NHANES demonstrated that the systemic immune-inflammation index significantly mediated the associations between multiple heavy metals and hepatic fibrosis markers, suggesting that metals may differentially regulate immune-inflammatory pathways to influence liver disease progression (61). In this context, dietary patterns with anti-inflammatory properties may modulate the relationship between metal exposure and liver health. The Mediterranean diet, characterized by high intake of fruits, vegetables, whole grains, and healthy fats, has been shown in multiple studies to enhance the body's capacity to mitigate heavy metal toxicity and, through its anti-inflammatory effects, to be associated with slower renal function decline, improved outcomes in arthritis, and reduced all-cause mortality in cancer patients (62–64). These findings provide a potential direction for future research on dietary interventions to ameliorate metal-induced liver injury. In addition, heavy metals inhibit lipoge nesis and enhance lipolysis in the adipose tissue by inducing incomplete autophagy (autophagy inhibition or autophagy overactivation), leading to NAFLD (8). Past studies have reported that heavy metal exposure is a risk factor for NAFLD and non-alcoholic steatohepatitis and that autophagy disorder is a potential mechanism of heavy metal-induced hepatotoxicity (65–67). Additionally, apoptosis, mitochondrial damage, epigenetic modifications, and endoplasmic reticulum stress have been reported to play roles in heavy metal-induced liver injury (6, 68–70). However, further research is required to elucidate the hepatotoxicity caused by heavy metal exposure.

This study identified Cd as the primary driver of liver function biomarkers through population-based cohort analysis, and further confirmed that Cd exposure can induce liver injury using an animal model. Recent studies have elucidated multiple synergistic mechanisms underlying Cd-induced hepatotoxicity, with lipid metabolism disorder emerging as a potential mechanism (71). Research has reported that Cd exacerbates liver injury by remodeling ceramide metabolism and promoting oxidative stress in hepatocytes (72). This finding is consistent with our transcriptomic results, which revealed significant enrichment of lipid metabolism-related pathways, supporting lipid metabolism disorder may play a potential role in Cd-induced liver injury, but its precise mechanism needs experimental validation. Furthermore, mitochondrial dysfunction represents a critical event in Cd-induced hepatotoxicity. Cd rapidly accumulates in mitochondria, directly inhibiting electron transport chain complexes, leading to sustained ROS production and mitochondrial membrane potential collapse (73). Recent advances have further confirmed that programmed cell death is a key downstream consequence of Cd-induced oxidative stress and mitochondrial dysfunction. Cd hepatotoxicity involves multiple cell death modalities, including apoptosis, autophagy, ferroptosis, and pyroptosis (74). The complexity of Cd-induced liver injury arises from the intricate interplay among these various mechanisms, suggesting that multi-target interventions may be necessary to achieve effective prevention or treatment.

Although sex-stratified analyses were conducted to explore potential effect modification, we did not observe statistically significant differences in the associations between heavy metal mixtures and liver function between males and females in our study populations. The absence of a pronounced gender effect may be related to age, and in middle aged and older adults, the protective hormonal effects in pre-menopausal women may be diminished, potentially reducing the disparity in susceptibility observed in younger populations. In addition, the chronic, low-level environmental exposure scenario in our study might affect both sexes more uniformly compared to high-dose occupational settings. While this lack of modification by gender is an important finding itself, indicating a general population health risk, future studies with larger sample sizes or focusing on specific life stages are warranted to confirm this observation.

Reliable exposure assessment is crucial in epidemiological studies. In this study, we used concentrations of heavy metals in blood as biomarkers of internal exposure. Blood metal levels are widely regarded as reliable indicators of internal dose, as they reflect cumulative exposure from multiple sources and exhibit relatively low variability, often remaining stable over time. For metals with longer biological half-lives, such as Cd and Pb, blood concentrations can serve as reasonable proxies for chronic exposure and body burden (75). In contrast, for metals with shorter half-lives, such as As and Al, blood levels may primarily reflect recent exposure and are subject to greater temporal variability (76). Urinary metal concentrations are often considered indicative of long-term exposure and can provide valuable insights into excretion patterns, particularly for certain metals like As. However, they may be influenced by factors such as renal clearance, circadian rhythms, and external exposure (77). Despite these inherent limitations, a single blood measurement is generally considered to reasonably classify long-term exposure in epidemiological studies, particularly in populations residing in areas with relatively stable environmental contamination. Future research should more comprehensively take into account the half-lives, metabolic pathways, and target organ toxicity of heavy metals to better interpret the toxic effects reflected in different biological matrices.

This study had several notable strengths. First, this was the first study to focus on the potential effects of environmental heavy metal mixtures on liver function in middle-aged and older adults individuals in northwest China, thereby filling the research gap in this particular demographic. Second, we integrated multiple complementary statistical approaches, including multiple linear regression and three mixture models (BKMR, WQS, and Qgcomp). By cross-validating results and balancing the advantages and limitations of different methods, we minimized potential biases associated with single-model analysis, ensuring the robustness and comprehensiveness of our findings. Finally, the establishment of a sub-chronic Cd exposure rat model provided experimental evidence supporting the biological plausibility of the association between Cd and liver injury observed in the human cohort; additionally, transcriptome sequencing technology further deciphered the potential mechanisms underlying liver injury induced by long-term low-dose Cd exposure. This not only supported the epidemiological findings but also offered important scientific basis for the in-depth development of relevant basic research.

However, this study had several limitations. First, the cross-sectional nature of the analysis precludes causal inference. While we observed significant associations between heavy metal mixtures and liver function indices, the temporal relationship between exposure and outcome cannot be established with certainty. Therefore, our findings should be interpreted as revealing important associations rather than definitive causal relationships. Future prospective cohort studies with repeated exposure and outcome measurements are needed to confirm the causal direction and underlying biological pathways. Second, the mechanism underlying the effect of heavy metal exposure on liver function remains unclear. Although animal experiments have demonstrated that Cd exposure can induce hepatic histopathological alterations and significantly affect liver gene expression, it is important to note the substantial differences between the human cohort study and the animal experiment. The former pertains to associations between exposure to heavy metal mixtures at environmental levels and liver function biomarkers in a general population, whereas the latter employed a single-metal, high-dose exposure paradigm. These differences constitute a translational gap between the two lines of evidence, warranting caution when extrapolating findings from animal models to human populations. Third, although we adjusted for multiple potential confounders, residual confounding cannot be completely ruled out. Unmeasured or imperfectly measured factors may have influenced the observed associations. For example, detailed dietary information (including nutritional status, specific food components, and caloric intake), viral hepatitis status, diabetes/metabolic syndrome, physical activity levels, socioeconomic status, occupational exposure history, and use of medications or herbal supplements were not comprehensively collected in this study. These factors could potentially confound the relationship between metal exposure and liver function. Future studies with more detailed and systematic collection of these lifestyle and environmental exposure data are warranted to confirm our findings. Finally, the generalizability of our findings to other populations, age groups, or exposure settings may be limited. These limitations highlight the need for cautious interpretation of our results and underscore the importance of future longitudinal studies incorporating repeated exposure measures and more comprehensive covariate data to reduce these uncertainties.

## 5 Conclusion

In summary, this study demonstrates a significant association between blood heavy metal exposure and liver function indices among middle-aged and older adults individuals in Northwest China. By applying multiple statistical models, we revealed that co-exposure to high concentrations of heavy metal mixtures is linked to liver function biomarkers, and further identified Cd emerged as the metal most consistently and strongly implicated across all models and outcomes in the local population. These findings are supported by experimental evidence from a rat model, in which Cd exposure led to elevated serum liver function markers and induced histopathological alterations in the liver. Transcriptomic sequencing suggested that hepatic lipid metabolism pathways may be involved in Cd-induced liver injury. Our findings add to the growing evidence that environmental heavy metal exposure may influence liver function biomarkers, and future studies should focus on elucidating the molecular mechanisms underlying the predominant role of Cd and expanding biomonitoring efforts in similarly exposed populations.

## Data Availability

The original contributions presented in the study are included in the article/Supplementary material, further inquiries can be directed to the corresponding author.
