# Supplementary figures and images for: Association of heavy metal mixtures with liver function biomarkers: multi-model analysis identifies cadmium as the primary driver
Source: Front Public Health. 2026 Apr 28;14:1817191. doi: 10.3389/fpubh.2026.1817191 (PMC13161090; doi:10.3389/fpubh.2026.1817191)

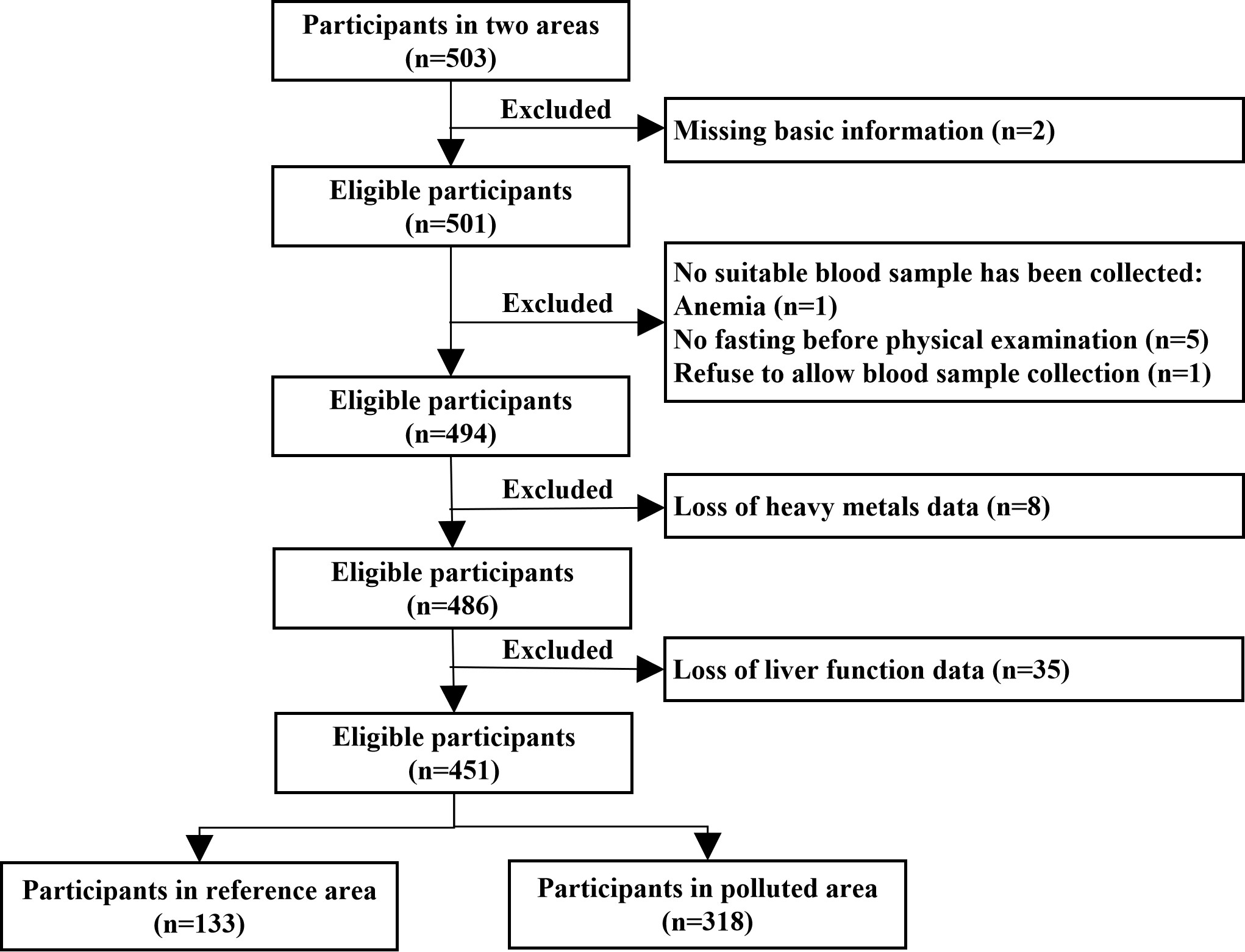

Supplement: Supplementary file 1 [file Image_1.JPEG]

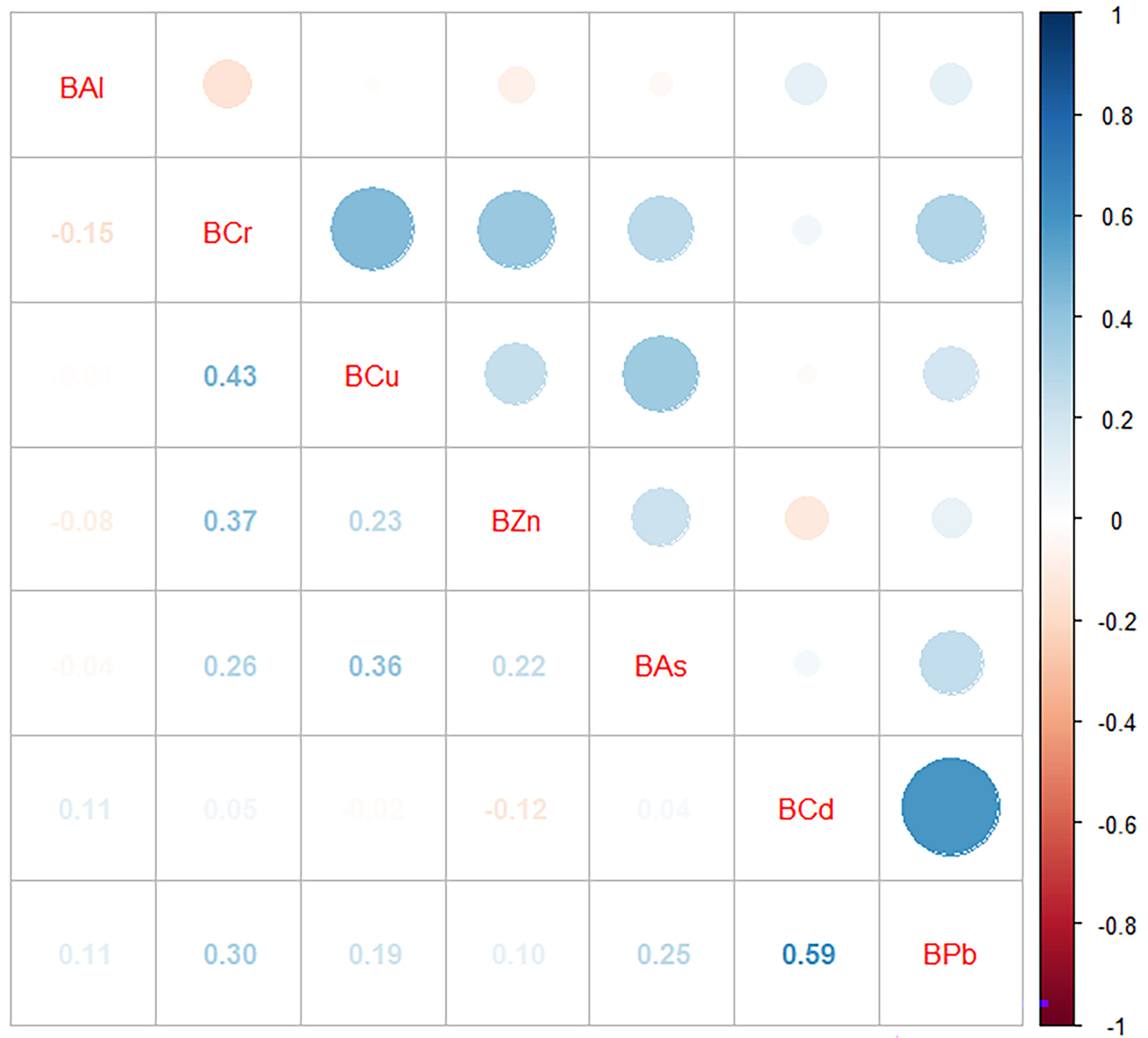

Supplement: Supplementary file 2 [file Image_2.JPEG]

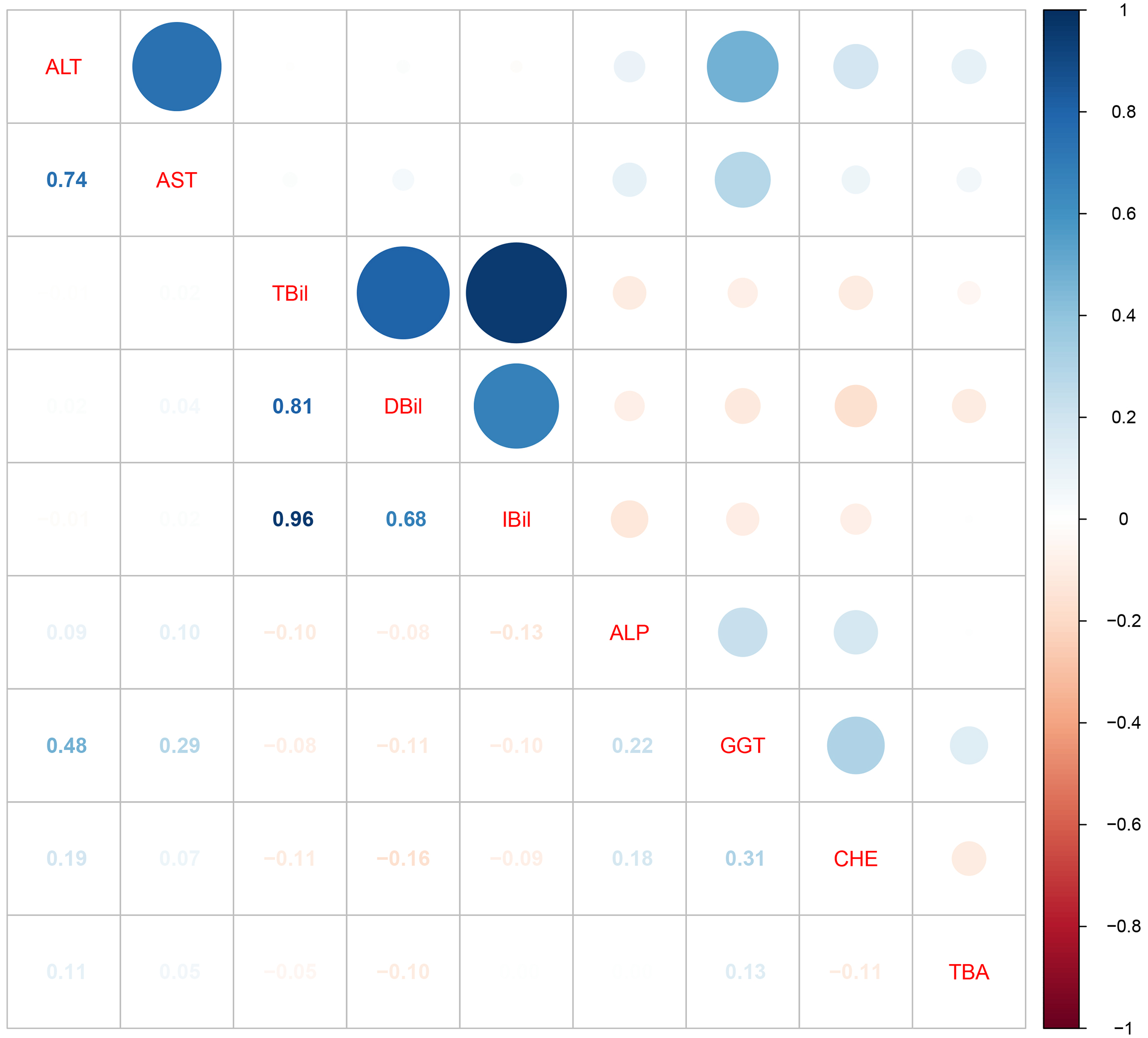

Supplement: Supplementary file 3 [file Image_3.JPEG]

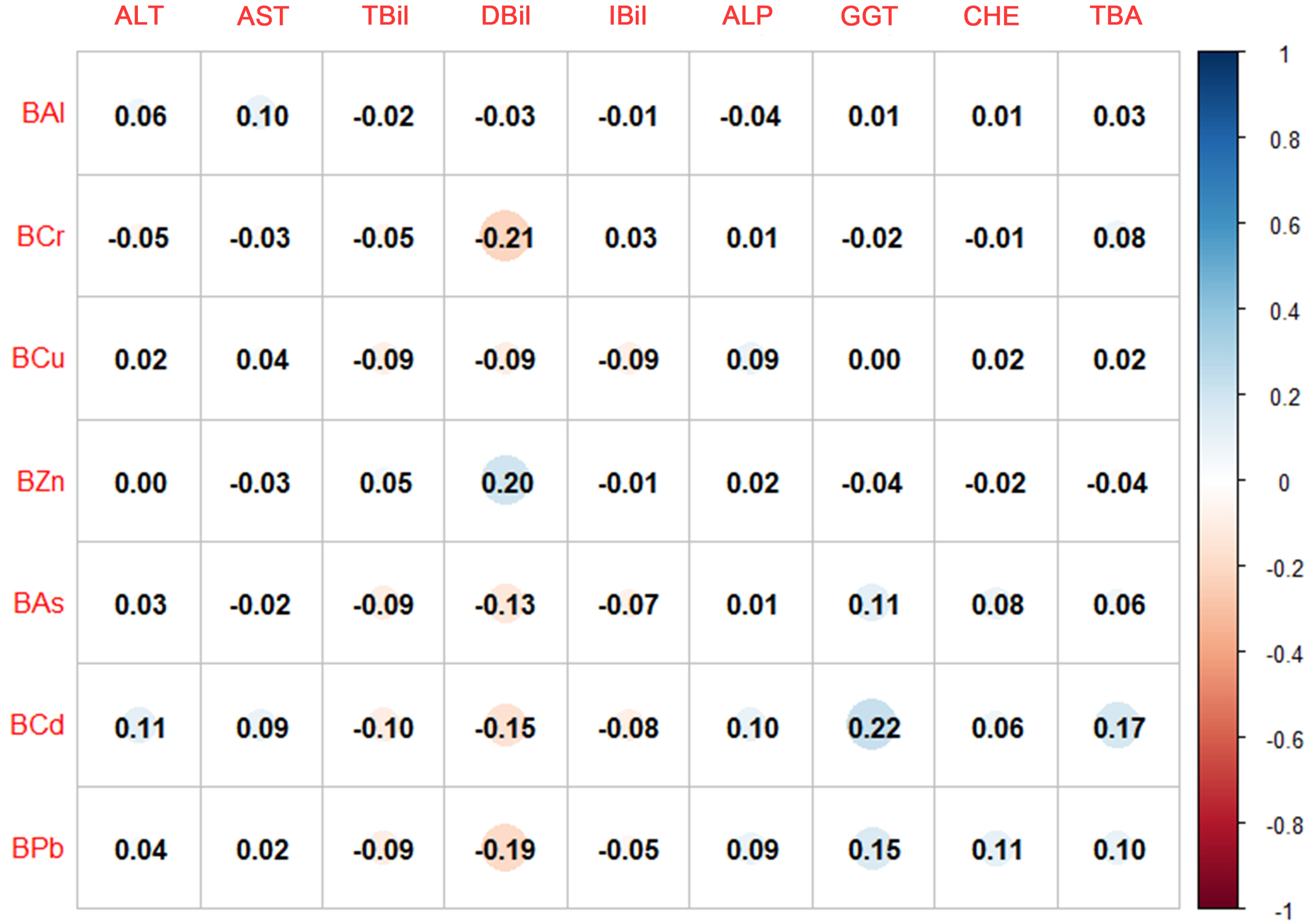

Supplement: Supplementary file 4 [file Image_4.JPEG]

**Graphical abstract**


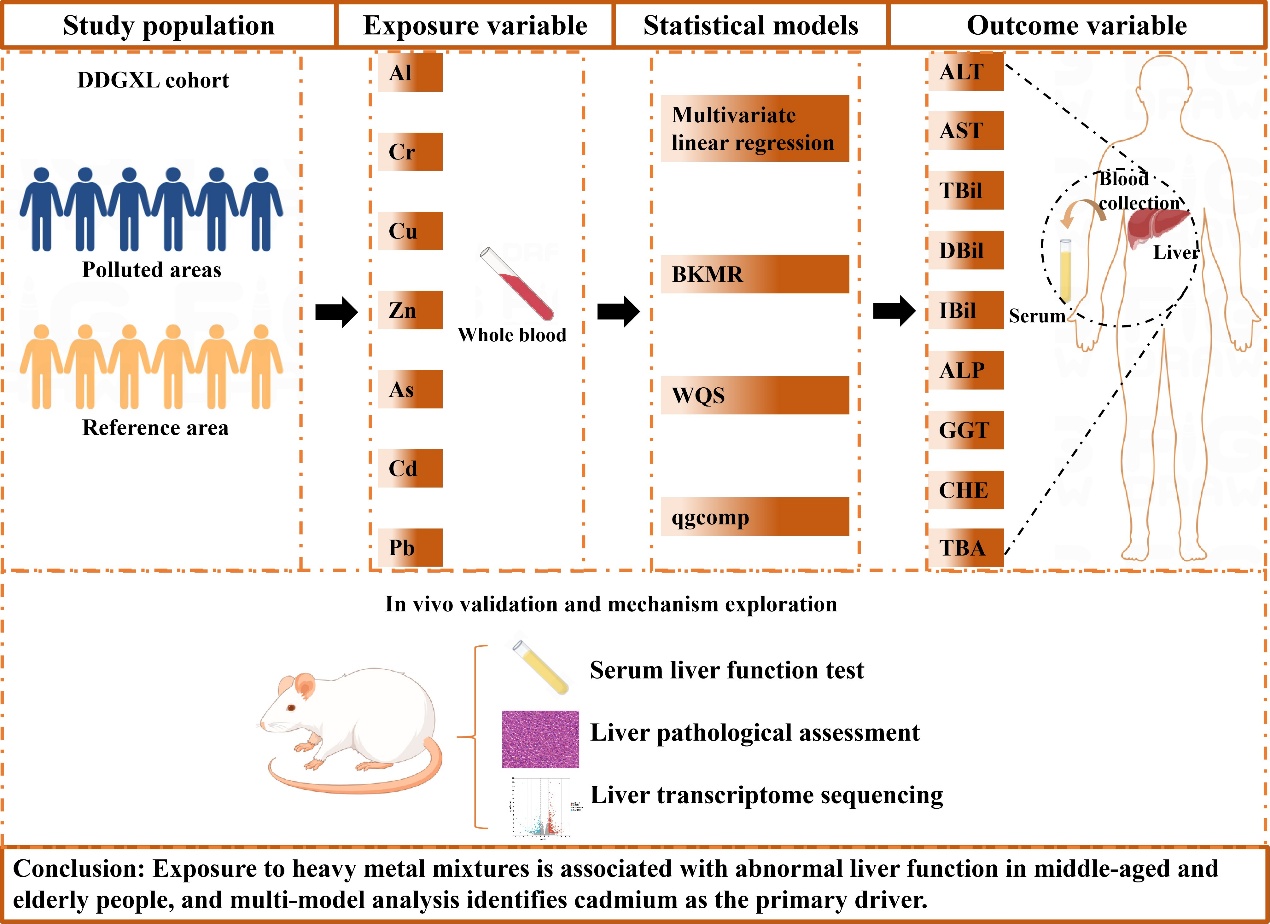

Supplement: Supplementary file 5 [file Table_1.DOCX]
