# Supplementary material for: Association of heavy metal mixtures with liver function biomarkers: multi-model analysis identifies cadmium as the primary driver
Source: Front Public Health. 2026 Apr 28;14:1817191. doi: 10.3389/fpubh.2026.1817191 (PMC13161090; doi:10.3389/fpubh.2026.1817191)
Supplement: Supplementary file 7 [file Table_3.DOCX]

| **Table 1.** General characteristics of the study population. | | | | |
| --- | --- | --- | --- | --- |
| Variable | All participants (*n* = 451) | Gender | | *P* Value |
|  |  | Male (*n* = 154) | Female (*n* = 297) |  |
| Age (year) | 56.12 (8.09) | 57.91 (7.13) | 55.20 (8.41) | 0.001** |
| Height (cm) | 161.42 (7.97) | 168.93 (5.90) | 157.53 (5.84) | <0.001** |
| Weight (kg) | 61.76 (9.96) | 66.35 (10.03) | 59.38 (9.07) | <0.001** |
| BMI (kg/m^2^) | 23.62 (3.44) | 23.21 (3.00) | 23.84 (3.63) | 0.065 |
| Smoking |  |  |  | <0.001** |
| Yes | 139 (30.8%) | 77 (50.0%) | 62 (20.9%) |  |
| No | 312 (69.2%) | 77 (50.0%) | 235 (79.1%) |  |
| Alcohol consumption | |  |  | 0.009** |
| Yes | 48 (10.6%) | 25 (16.2%) | 23 (7.7%) |  |
| No | 403 (89.4%) | 129 (83.8%) | 274 (92.3%) |  |
| Tea drinking |  |  |  | <0.001** |
| Yes | 194 (43.0%) | 84 (54.5%) | 110 (37.0%) |  |
| No | 257 (57.0%) | 70 (45.5%) | 187 (63.0%) |  |
| BAl (μg/L) | 185.12 (24.34, 474.84) | 163.30 (13.32, 445.13) | 194.81 (26.39, 494.76) | 0.500 |
| BCr (μg/L) | 122.05 (69.44, 137.80) | 121.48 (72.20, 137.45) | 122.40 (69.29, 137.90) | 0.789 |
| BCu (μg/L) | 826.84 (722.22, 929.50) | 803.19 (696.76, 916.82) | 838.58 (726.30, 938.07) | 0.065 |
| BZn (μg/L) | 5549.23 (4719.60, 6231.11) | 5589.31 (4853.06, 6427.90) | 5543.82 (4560.27, 6152.05) | 0.080 |
| BAs (μg/L) | 8.50 (3.69, 10.81) | 8.68 (5.60, 11.15) | 8.30 (3.07, 10.70) | 0.092 |
| BCd (μg/L) | 0.67 (0.08, 4.01) | 1.21 (0.08, 5.02) | 0.42 (0.08, 3.17) | 0.009** |
| BPb (μg/L) | 21.62 (11.67, 36.23) | 23.62 (12.66, 39.09) | 20.90 (11.07, 34.74) | 0.077 |
| ALT (U/L) | 21.10 (15.50, 30.20) | 23.25 (16.40, 31.95) | 20.80 (16.85, 29.00) | 0.175 |
| AST (U/L) | 23.80 (20.20, 28.90) | 23.30 (19.98, 28.25) | 24.30 (20.35, 29.05) | 0.276 |
| TBil (μmol/L) | 17.00 (13.80, 21.70) | 18.45 (14.88, 22.90) | 16.30 (13.33, 20.70) | 0.002** |
| DBil (μmol/L) | 6.10 (4.80, 7.20) | 6.50 (5.20, 7.73) | 5.80 (4.70, 6.85) | 0.001** |
| IBil (μmol/L) | 11.20 (8.70, 14.60) | 12.00 (9.58, 15.83) | 10.80 (8.45, 14.05) | 0.011* |
| ALP (U/L) | 90.00 (76.00, 108.00) | 90.00 (78.00, 105.25) | 91.00 (74.00, 108.50) | 0.671 |
| GGT (U/L) | 17.00 (13.00, 27.00) | 21.00 (15.75, 31.00) | 16.00 (12.00, 24.00) | <0.001** |
| CHE (KU/L) | 8.85 (7.80, 10.02) | 8.49 (7.50, 9.54) | 9.07 (7.95, 10.18) | 0.004** |
| TBA (μmol/L) | 1.90 (1.40, 2.90) | 1.90 (1.50, 3.20) | 1.90 (1.30, 2.80) | 0.175 |
| BMI, body mass index; BAl, aluminum in blood; BCr, chromium in blood; BCu, cuprum in blood; BZn; zinc in blood; BAs, arsenic in blood; BCd, cadmium in blood; BPb, lead in blood; ALT, alanine aminotransferase; AST, aspartate aminotransferase; TBil, total bilirubin; DBil, direct bilirubin; IBil, indirect bilirubin; ALP, alkaline phosphatase; GGT, gamma glutamyl transpeptidase; CHE, cholinesterase; TBA, total bile acid.  Data were presented as n (%) for categorical data, mean (standard deviation) for normal distribution data, and median (interquartile range) for abnormal distribution data.  Student’s t-test or Mann-Whitney U tests were used for comparison of the continuous variables according to the data distribution, and Chi-square test for the categorical variables.  ***P* < 0.01; **P* < 0.05. | | | | |
