# Supplementary material for: Association of heavy metal mixtures with liver function biomarkers: multi-model analysis identifies cadmium as the primary driver
Source: Front Public Health. 2026 Apr 28;14:1817191. doi: 10.3389/fpubh.2026.1817191 (PMC13161090; doi:10.3389/fpubh.2026.1817191)

**Supplementary figures**

**Association of heavy metal mixtures with abnormal liver function: Multi-model analysis identifies cadmium as the primary driver**

Honglong Zhang^1, 2^, Xingwang Zhu^2^, Meng Tian^2, 7^, Tingting Wang^2^, Ruipeng Wang^2^, Mingtong Zhang^8^, Jun Yan^2,3,4,5 *^, Xun Li^2,3,4,5,6^

^1^ Department of Breast and Thyroid Surgery, Union Hospital, Tongji Medical College, Huazhong University of Science and Technology, Wuhan, 430022, China.

^2^ The First School of Clinical Medicine, Lanzhou University, Lanzhou 730000, PR China

^3^ Department of General Surgery, The First Hospital of Lanzhou University, Lanzhou 730000, PR China

^4^ Hepatopancreatobiliary Surgery Institute of Gansu Province, Lanzhou 730000, PR China

^5^ Key Laboratory of Biotherapy and Regenerative Medicine of Gansu Province, Lanzhou 730000, PR China

^6^ Clinical Research Center for General Surgery of Gansu Province, Lanzhou 730000, PR China

^7^ Deyang People’s Hospital, Deyang 618000, PR China

^8^ Gansu Provincial Institute of Drug Control, Lanzhou 730000, PR China

^*^Corresponding author: Jun Yan. Department of General Surgery, The First Hospital of Lanzhou University, No.1 Donggang West Road, Chengguan District 730030, Lanzhou, Gansu, China. Tel: +86-13919840487. Email: ldyysys@126.com

**
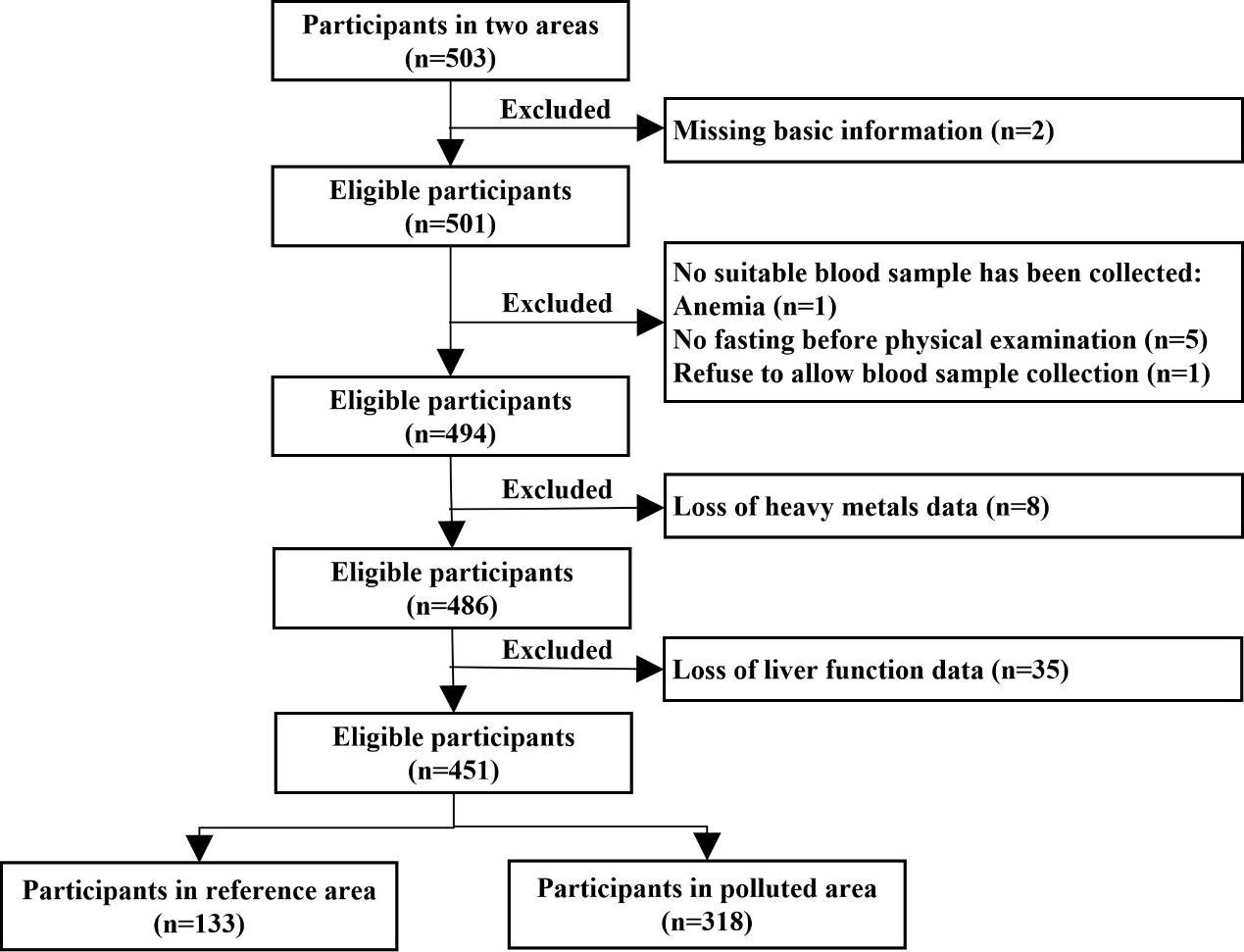
Fig. S1.** Flow chart of participants finally included in this study (n = 451).


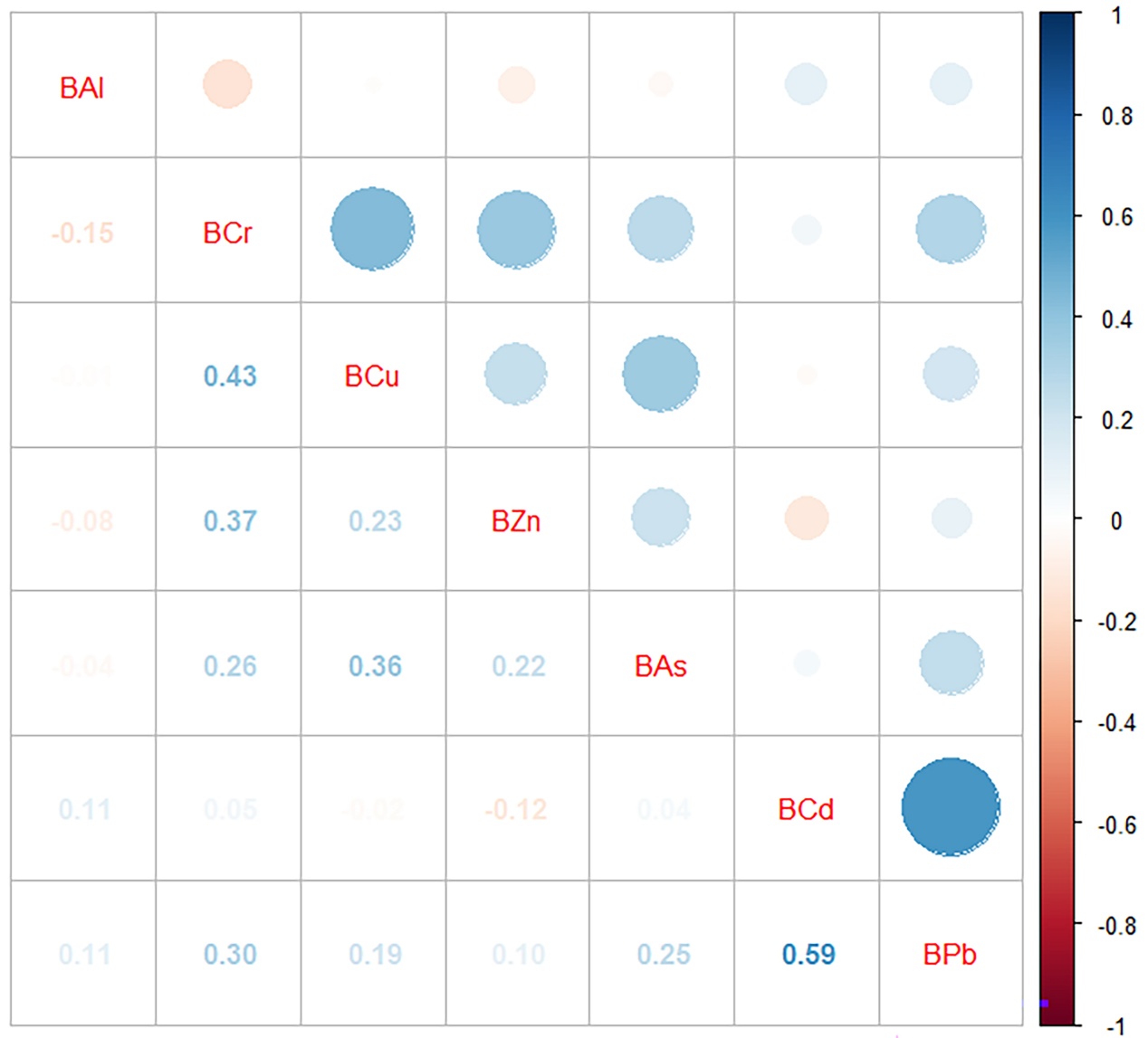
**Fig. S2.** Spearman correlation plot for blood heavy metals of participants (*n* = 541). **Abbreviations:** BAl, aluminum in blood; BCr, chromium in blood; BCu, cuprum in blood; BZn; zinc in blood; BAs, arsenic in blood; BCd, cadmium in blood; BPb, lead in blood. Heavy metals in blood were log transformed.

**Fig. S3.** Spearman correlation plot for liver function indices of participants (*n* = 541). **Abbreviations:** ALT, alanine aminotransferase; AST, aspartate aminotransferase; TBil, total bilirubin; DBil, direct bilirubin; IBil, indirect bilirubin; ALP, alkaline phosphatase; GGT, gamma glutamyl transpeptidase; CHE, cholinesterase; TBA, total bile acid. Liver function indices were log transformed.
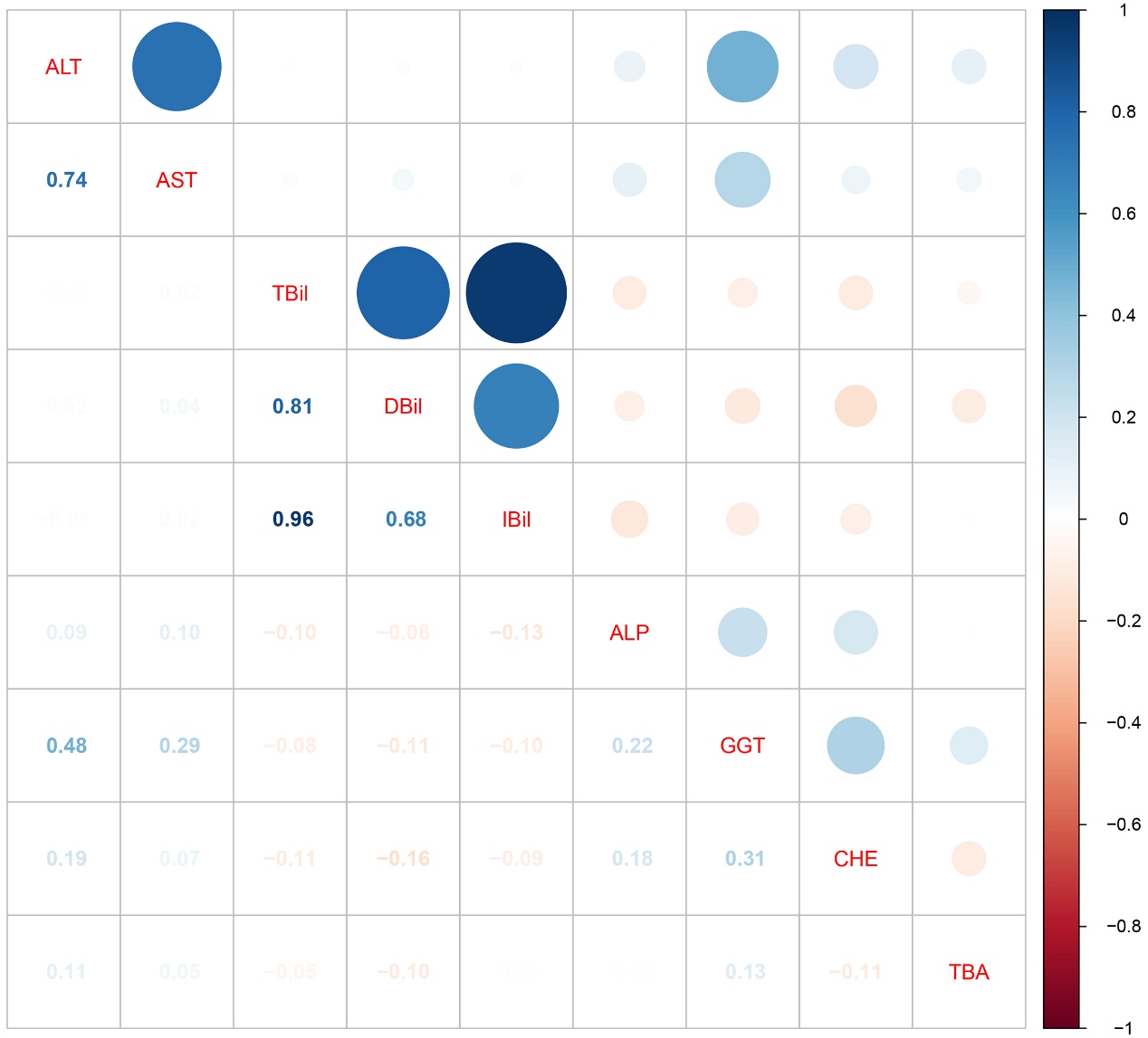


**Fig. S4.** Spearman correlation plot for blood heavy metals and liver function indices of participants (*n* = 541). **Abbreviations:** BAl, aluminum in blood; BCr, chromium in blood; BCu, cuprum in blood; BZn; zinc in blood; BAs, arsenic in blood; BCd, cadmium in blood; BPb, lead in blood ; ALT, alanine aminotransferase; AST, aspartate aminotransferase; TBil, total bilirubin; DBil, direct bilirubin; IBil, indirect bilirubin; ALP, alkaline phosphatase; GGT, gamma glutamyl transpeptidase; CHE, cholinesterase; TBA, total bile acid. Heavy metals in blood and liver function indices were log transformed.
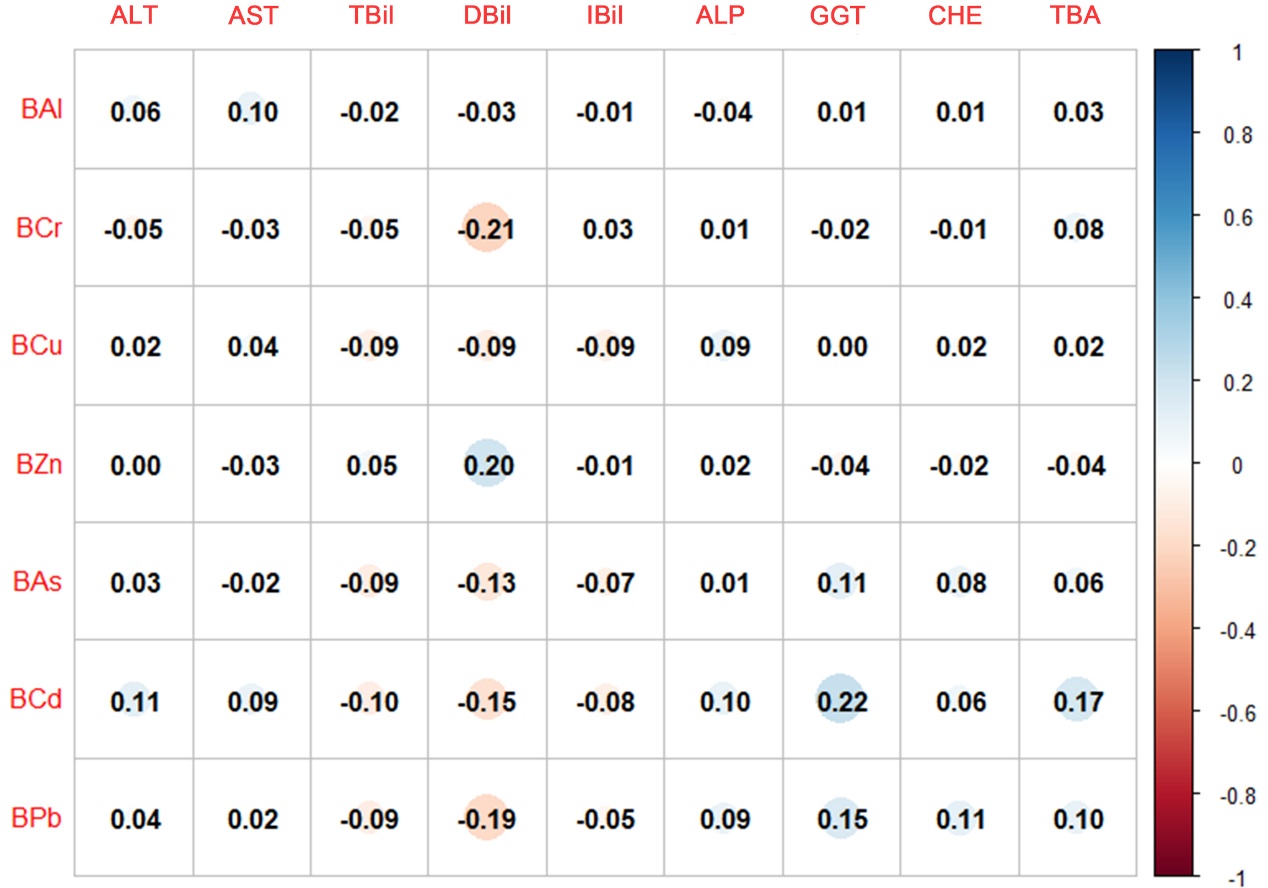

Supplement: Supplementary file 8 [file Table_4.DOCX]
