# Supplementary material for: Association of heavy metal mixtures with liver function biomarkers: multi-model analysis identifies cadmium as the primary driver
Source: Front Public Health. 2026 Apr 28;14:1817191. doi: 10.3389/fpubh.2026.1817191 (PMC13161090; doi:10.3389/fpubh.2026.1817191)
Supplement: Supplementary file 9 [file Table_5.DOCX]

**Supplementary tables**

**Association of heavy metal mixtures with abnormal liver function: Multi-model analysis identifies cadmium as the primary driver**

Honglong Zhang^1, 2^, Xingwang Zhu^2^, Meng Tian^2, 7^, Tingting Wang^2^, Ruipeng Wang^2^, Mingtong Zhang^8^, Jun Yan^2,3,4,5 *^, Xun Li^2,3,4,5,6^

^1^ Department of Breast and Thyroid Surgery, Union Hospital, Tongji Medical College, Huazhong University of Science and Technology, Wuhan, 430022, China.

^2^ The First School of Clinical Medicine, Lanzhou University, Lanzhou 730000, PR China

^3^ Department of General Surgery, The First Hospital of Lanzhou University, Lanzhou 730000, PR China

^4^ Hepatopancreatobiliary Surgery Institute of Gansu Province, Lanzhou 730000, PR China

^5^ Key Laboratory of Biotherapy and Regenerative Medicine of Gansu Province, Lanzhou 730000, PR China

^6^ Clinical Research Center for General Surgery of Gansu Province, Lanzhou 730000, PR China

^7^ Deyang People’s Hospital, Deyang 618000, PR China

^8^ Gansu Provincial Institute of Drug Control, Lanzhou 730000, PR China

^*^Corresponding author: Jun Yan. Department of General Surgery, The First Hospital of Lanzhou University, No.1 Donggang West Road, Chengguan District 730030, Lanzhou, Gansu, China. Tel: +86-13919840487. Email: ldyysys@126.com

| **Table S1.** Linearity and precision data obtained for blood heavy metals. | | | | | |  | | | |
| --- | --- | --- | --- | --- | --- | --- | --- | --- | --- |
| Variable | Linear regression equation | LOD | Recovery rate (%) | RSD (%) | GM | Mean | Percentile | | |
|  |  |  |  |  |  |  | 25th | 50th | 75th |
| BAl (ng/mL) | y = 0.3744x-0.0214 | 0.50 | 96.5–99.4 | 0.37–3.20 | 105.60 | 449.91 | 24.34 | 185.12 | 474.84 |
| BCr (ng/mL) | y = 0.8328x+0.1070 | 0.30 | 99.1–105.3 | 1.34–5.32 | 84.55 | 106.43 | 69.44 | 122.05 | 137.80 |
| BCu (ng/mL) | y = 4.438x+0.3297 | 1.00 | 100.1–110.2 | 1.89–4.23 | 762.33 | 798.83 | 722.22 | 826.84 | 929.50 |
| BZn (ng/mL) | y = 2.4381x+0.4337 | 5.00 | 94.6–103.8 | 3.08–6.43 | 5336.05 | 5497.51 | 4719.60 | 5549.23 | 6231.11 |
| BAs (ng/mL) | y = 0.0322x-0.0033 | 0.50 | 98.8–102.6 | 1.33–5.29 | 5.04 | 7.84 | 3.69 | 8.50 | 10.81 |
| BCd (ng/mL) | y = 0.0586x-0.0004 | 0.08 | 98.3–101.5 | 1.30–3.16 | 0.64 | 2.46 | 0.08 | 0.67 | 4.01 |
| BPb (ng/mL) | y = 0.2671x+0.027 | 0.11 | 95.8–96.4 | 1.53–2.03 | 16.99 | 25.52 | 11.67 | 21.62 | 36.23 |
| BAl: aluminum in blood; BCr: chromium in blood; BCu: cuprum in blood; BZn: zinc in blood; BAs: arsenic in blood; BCd: cadmium in blood; BPb: lead in blood; LOD, limit of detection; RSD, relative standard deviation; GM: geomean. | | | | | | | | | |

| **Table S2.** Normality test of heavy metals in blood and liver function parameters. | | | | |
| --- | --- | --- | --- | --- |
| Variable | Kolmogorov-Smirnova | | Shapiro-Wilk | |
|  | *Z* | *P* Value | *W* | *P* Value |
| BAl | 0.298 | <0.001** | 0.523 | <0.001** |
| BCr | 0.167 | <0.001** | 0.914 | <0.001** |
| BCu | 0.103 | <0.001** | 0.934 | <0.001** |
| BZn | 0.036 | 0.194 | 0.994 | 0.055 |
| BAs | 0.081 | <0.001** | 0.896 | <0.001** |
| BCd | 0.239 | <0.001** | 0.747 | <0.001** |
| BPb | 0.093 | <0.001** | 0.935 | <0.001** |
| ALT | 0.173 | <0.001** | 0.734 | <0.001** |
| AST | 0.175 | <0.001** | 0.648 | <0.001** |
| TBil | 0.100 | <0.001** | 0.921 | <0.001** |
| DBil | 0.090 | <0.001** | 0.867 | <0.001** |
| IBil | 0.109 | <0.001** | 0.821 | <0.001** |
| ALP | 0.176 | <0.001** | 0.487 | <0.001** |
| GGT | 0.252 | <0.001** | 0.487 | <0.001** |
| CHE | 0.095 | <0.001** | 0.938 | <0.001** |
| TBA | 0.224 | <0.001** | 0.565 | <0.001** |
| BAl, aluminum in blood; BCr, chromium in blood; BCu, cuprum in blood; BZn; zinc in blood; BAs, arsenic in blood; BCd, cadmium in blood; BPb, lead in blood; ALT, alanine aminotransferase; AST, aspartate aminotransferase; TBil, total bilirubin; DBil, direct bilirubin; IBil, indirect bilirubin; ALP, alkaline phosphatase; GGT, gamma glutamyl transpeptidase; CHE, cholinesterase; TBA, total bile acid.  The Kolmogorov-Smirnova test and the Shapiro-Wilk test were used to verify that the data is normally distributed.  ***P* < 0.01; **P* < 0.05 | | | | |

| **Table S3.** Correlation analysis of heavy metals in blood and liver function parameters. | | | | | | |  | | |  |  | |  | |  |
| --- | --- | --- | --- | --- | --- | --- | --- | --- | --- | --- | --- | --- | --- | --- | --- |
| Metals |  | ALT | AST | TBil | DBil | IBil | | ALP | GGT | | | CHE | | TBA | |
| BAl | *r* | 0.064 | 0.097 | -0.018 | -0.030 | -0.012 | | -0.038 | 0.013 | | | 0.008 | | 0.030 | |
|  | *P* | 0.175 | 0.040* | 0.699 | 0.524 | 0.793 | | 0.416 | 0.777 | | | 0.869 | | 0.520 | |
| BCr | *r* | -0.053 | -0.034 | -0.050 | -0.216 | 0.027 | | 0.010 | -0.015 | | | -0.008 | | 0.078 | |
|  | *P* | 0.259 | 0.478 | 0.287 | <0.001** | 0.574 | | 0.833 | 0.749 | | | 0.858 | | 0.096 | |
| BCu | *r* | 0.018 | 0.042 | -0.085 | -0.092 | -0.086 | | 0.088 | 0.003 | | | 0.024 | | 0.024 | |
|  | *P* | 0.710 | 0.379 | 0.071 | 0.052 | 0.067 | | 0.063 | 0.952 | | | 0.606 | | 0.604 | |
| BZn | *r* | 0.004 | -0.028 | 0.055 | 0.197 | -0.008 | | 0.015 | -0.041 | | | -0.024 | | -0.039 | |
|  | *P* | 0.926 | 0.555 | 0.248 | <0.001** | 0.862 | | 0.750 | 0.384 | | | 0.608 | | 0.405 | |
| BAs | *r* | 0.031 | -0.016 | -0.095 | -0.130 | -0.073 | | 0.011 | 0.112 | | | 0.084 | | 0.063 | |
|  | *P* | 0.507 | 0.731 | 0.044* | 0.006** | 0.120 | | 0.812 | 0.017* | | | 0.075 | | 0.184 | |
| BCd | *r* | 0.112 | 0.088 | -0.098 | -0.154 | -0.083 | | 0.096 | 0.221 | | | 0.062 | | 0.173 | |
|  | *P* | 0.018* | 0.062 | 0.037* | 0.001** | 0.079 | | 0.041* | <0.001** | | | 0.191 | | <0.001** | |
| BPb | *r* | 0.041 | 0.019 | -0.094 | -0.194 | -0.047 | | 0.089 | 0.152 | | | 0.106 | | 0.098 | |
|  | *P* | 0.387 | 0.691 | 0.046* | <0.001** | 0.322 | | 0.059 | 0.001** | | | 0.024* | | 0.037* | |
| BAl, aluminum in blood; BCr, chromium in blood; BCu, cuprum in blood; BZn; zinc in blood; BAs, arsenic in blood; BCd, cadmium in blood; BPb, lead in blood; ALT, alanine aminotransferase; AST, aspartate aminotransferase; TBil, total bilirubin; DBil, direct bilirubin; IBil, indirect bilirubin; ALP, alkaline phosphatase; GGT, gamma glutamyl transpeptidase; CHE, cholinesterase; TBA, total bile acid.  Heavy metals in blood and liver function parameters were log transformed.  Spearman correlation analysis was used to test the correlation between heavy metals and biomarkers. r > 0 means positive correlation, r < 0 means negative correlation.  **P < 0.01; *P < 0.05 | | | | | | | | | | | | | | | |

| **Table S4.** The relationship between heavy metals in blood and liver function parameters. | | | | | | | | | | | | | | | | | | | | | |
| --- | --- | --- | --- | --- | --- | --- | --- | --- | --- | --- | --- | --- | --- | --- | --- | --- | --- | --- | --- | --- | --- |
| Liver function parameters | | | Metals | | | Model 1 | | | | | | | | Model 2 | | | | | | | |
|  |  |  |  |  |  | *β (95% CI)* | | | | | *P* Value | | | *β (95% CI)* | | | | | *P* Value | | |
| ALT | | | BAl | | | 0.057 (-0.035, 0.150) | | | | | 0.224 | | | 0.043 (-0.049, 0.135) | | | | | 0.357 | | |
|  |  |  | BCr | | | -0.058 (-0.151, 0.035) | | | | | 0.218 | | | -0.049 (-0.142, 0.044) | | | | | 0.299 | | |
|  |  |  | BCu | | | 0.022 (-0.071, 0.115) | | | | | 0.640 | | | 0.042 (-0.050, 0.135) | | | | | 0.366 | | |
|  |  |  | BZn | | | 0.008 (-0.085, 0.101) | | | | | 0.863 | | | 0.011 (-0.081, 0.103) | | | | | 0.819 | | |
|  |  |  | BAs | | | 0.041 (-0.055, 0.137) | | | | | 0.397 | | | 0.046 (-0.050, 0.141) | | | | | 0.346 | | |
|  |  |  | BCd | | | 0.131 (0.028, 0.233) | | | | | 0.012* | | | 0.142 (0.040, 0.244) | | | | | 0.007** | | |
|  |  |  | BPb | | | 0.052 (-0.042, 0.146) | | | | | 0.282 | | | 0.062 (-0.032, 0.156) | | | | | 0.195 | | |
| AST | | | BAl | | | 0.093 (0.000, 0.185) | | | | | 0.049* | | | 0.086 (-0.008, 0.179) | | | | | 0.072 | | |
|  |  |  | BCr | | | -0.050 (-0.143, 0.043) | | | | | 0.290 | | | -0.042 (-0.137, 0.052) | | | | | 0.378 | | |
|  |  |  | BCu | | | 0.047 (-0.046, 0.139) | | | | | 0.321 | | | 0.048 (-0.046, 0.142) | | | | | 0.314 | | |
|  |  |  | BZn | | | -0.024 (-0.117, 0.069) | | | | | 0.612 | | | -0.016 (-0.110, 0.077) | | | | | 0.733 | | |
|  |  |  | BAs | | | -0.010 (-0.106, 0.087) | | | | | 0.846 | | | -0.002 (-0.099, 0.095) | | | | | 0.969 | | |
|  |  |  | BCd | | | 0.098 (-0.004, 0.201) | | | | | 0.061 | | | 0.114 (0.010, 0.218) | | | | | 0.0032* | | |
|  |  |  | BPb | | | 0.007 (-0.087, 0.101) | | | | | 0.890 | | | 0.021 (-0.075, 0.116) | | | | | 0.668 | | |
| TBil | | | BAl | | | 0.000 (-0.093, 0.092) | | | | | 0.996 | | | 0.010 (-0.082, 0.103) | | | | | 0.825 | | |
|  |  |  | BCr | | | -0.049 (-0.141, 0.043) | | | | | 0.298 | | | -0.061 (-0.153, 0.032) | | | | | 0.199 | | |
|  |  |  | BCu | | | -0.098 (-0.190, -0.005) | | | | | 0.038* | | | -0.092 (-0.185, -0.000) | | | | | 0.049* | | |
|  |  |  | BZn | | | 0.040 (-0.053, 0.133) | | | | | 0.396 | | | 0.025 (-0.068, 0.117) | | | | | 0.599 | | |
|  |  |  | BAs | | | -0.101 (-0.196, -0.005) | | | | | 0.039* | | | -0.113 (-0.208, -0.018) | | | | | 0.020* | | |
|  |  |  | BCd | | | -0.133 (-0.236, -0.031) | | | | | 0.010* | | | -0.166 (-0.268, -0.064) | | | | | 0.001** | | |
|  |  |  | BPb | | | -0.119 (-0.212, -0.025) | | | | | 0.013* | | | -0.149 (-0.242, -0.056) | | | | | 0.002** | | |
| DBil | | | BAl | | | -0.017 (-0.110, 0.076) | | | | | 0.718 | | | -0.006 (-0.099, 0.087) | | | | | 0.896 | | |
|  |  |  | BCr | | | -0.203 (-0.294, -0.112) | | | | | <0.001** | | | -0.217 (-0.308, -0.126) | | | | | <0.001** | | |
|  |  |  | BCu | | | -0.070 (-0.163, 0.022) | | | | | 0.135 | | | -0.065 (-0.158, 0.027) | | | | | 0.168 | | |
|  |  |  | BZn | | | 0.168 (0.077, 0.260) | | | | | <0.001** | | | 0.152 (0.061, 0.244) | | | | | 0.001** | | |
|  |  |  | BAs | | | -0.125 (-0.220, -0.030) | | | | | 0.010* | | | -0.141 (-0.236, -0.046) | | | | | 0.004** | | |
|  |  |  | BCd | | | -0.172 (-0.274, -0.070) | | | | | 0.001** | | | -0.207 (-0.309, -0.105) | | | | | <0.001** | | |
|  |  |  | BPb | | | -0.207 (-0.299, -0.115) | | | | | <0.001** | | | -0.238 (-0.330, -0.146) | | | | | <0.001** | | |
| IBil | | | BAl | | | 0.006 (-0.087, 0.099) | | | | | 0.901 | | | 0.012 (-0.080, 0.105) | | | | | 0.797 | | |
|  |  |  | BCr | | | 0.029 (-0.064, 0.122) | | | | | 0.540 | | | 0.022 (-0.071, 0.115) | | | | | 0.640 | | |
|  |  |  | BCu | | | -0.101 (-0.194, -0.009) | | | | | 0.031* | | | -0.095 (-0.187, -0.003) | | | | | 0.043* | | |
|  |  |  | BZn | | | -0.014 (-0.107, 0.078) | | | | | 0.760 | | | -0.026 (-0.118, 0.067) | | | | | 0.586 | | |
|  |  |  | BAs | | | -0.073 (-0.169, 0.022) | | | | | 0.133 | | | -0.079 (-0.175, 0.016) | | | | | 0.104 | | |
|  |  |  | BCd | | | -0.130 (-0.233, -0.028) | | | | | 0.013* | | | -0.157 (-0.260, -0.055) | | | | | 0.003* | | |
|  |  |  | BPb | | | -0.068 (-0.162, 0.026) | | | | | 0.157 | | | -0.093 (-0.187, 0.000) | | | | | 0.051 | | |
| ALP | | | BAl | | | -0.012 (-0.104, -0.081) | | | | | 0.807 | | | -0.017 (-0.108, 0.074) | | | | | 0.713 | | |
|  |  |  | BCr | | | 0.004 (-0.089, 0.097) | | | | | 0.935 | | | 0.014 (-0.078, 0.106) | | | | | 0.767 | | |
|  |  |  | BCu | | | 0.078 (-0.015, 0.170) | | | | | 0.098 | | | 0.076 (-0.014, 0.167) | | | | | 0.099 | | |
|  |  |  | BZn | | | 0.020 (-0.072, 0.113) | | | | | 0.665 | | | 0.030 (-0.061, 0.121) | | | | | 0.513 | | |
|  |  |  | BAs | | | 0.022 (-0.074, 0.118) | | | | | 0.656 | | | 0.039 (-0.055, 0.134) | | | | | 0.412 | | |
|  |  |  | BCd | | | 0.107 (0.005, 0.210) | | | | | 0.040* | | | 0.101 (-0.001, 0.202) | | | | | 0.051 | | |
|  |  |  | BPb | | | 0.098 (0.005, 0.192) | | | | | 0.040* | | | 0.098 (0.005, 0.190) | | | | | 0.038* | | |
| GGT | | | BAl | | | 0.000 (-0.092, 0.092) | | | | | 0.999 | | | 0.017 (-0.106, 0.071) | | | | | 0.700 | | |
|  |  |  | BCr | | | 0.004 (-0.089, 0.096) | | | | | 0.940 | | | 0.011 (-0.077, 0.100) | | | | | 0.800 | | |
|  |  |  | BCu | | | 0.006 (-0.086, 0.098) | | | | | 0.899 | | | 0.049 (-0.039, 0.138) | | | | | 0.274 | | |
|  |  |  | BZn | | | 0.000 (-0.093, 0.092) | | | | | 0.992 | | | -0.001 (-0.089, 0.088) | | | | | 0.989 | | |
|  |  |  | BAs | | | 0.127 (0.032, 0.222) | | | | | 0.009** | | | 0.133 (0.042, 0.224) | | | | | 0.004** | | |
|  |  |  | BCd | | | 0.243 (0.143, 0.343) | | | | | <0.001** | | | 0.236 (0.140, 0.333) | | | | | <0.001** | | |
|  |  |  | BPb | | | 0.160 (0.067, 0.252) | | | | | 0.001** | | | 0.157 (0.068, 0.246) | | | | | 0.001** | | |
| CHE | | | BAl | | | -0.006 (-0.099, 0.087) | | | | | 0.900 | | | -0.025 (-0.116, 0.067) | | | | | 0.597 | | |
|  |  |  | BCr | | | -0.001 (0.094, 0.092) | | | | | 0.976 | | | 0.009 (-0.082, 0.101) | | | | | 0.842 | | |
|  |  |  | BCu | | | 0.030 (-0.063, 0.123) | | | | | 0.523 | | | 0.035 (-0.056, 0.126) | | | | | 0.452 | | |
|  |  |  | BZn | | | 0.005 (-0.087, 0.098) | | | | | 0.908 | | | 0.028 (-0.063, 0.119) | | | | | 0.541 | | |
|  |  |  | BAs | | | 0.092 (-0.003, 0.188) | | | | | 0.059 | | | 0.119 (0.025, 0.213) | | | | | 0.013* | | |
|  |  |  | BCd | | | 0.070 (-0.033, 0.173) | | | | | 0.182 | | | 0.096 (-0.005, 0.197) | | | | | 0.063 | | |
|  |  |  | BPb | | | 0.083 (-0.011, 0.176) | | | | | 0.085 | | | 0.100 (0.008, 0.192) | | | | | 0.034* | | |
| TBA | | | BAl | | | 0.005 (-0.087, 0.098) | | | | | 0.909 | | | -0.006 (-0.098, 0.086) | | | | | 0.900 | | |
|  |  |  | BCr | | | 0.077 (-0.016, 0.169) | | | | | 0.103 | | | 0.092 (0.000, 0.185) | | | | | 0.050* | | |
|  |  |  | BCu | | | 0.009 (-0.083, 0.102) | | | | | 0.192 | | | 0.024 (-0.068, 0.116) | | | | | 0.608 | | |
|  |  |  | BZn | | | -0.059 (-0.152, 0.033) | | | | | 0.207 | | | -0.055 (-0.147, 0.037) | | | | | 0.244 | | |
|  |  |  | BAs | | | 0.056 (-0.040, 0.151) | | | | | 0.254 | | | 0.068 (-0.028, 0.163) | | | | | 0.163 | | |
|  |  |  | BCd | | | 0.169 (0.068, 0.270) | | | | | 0.001** | | | 0.162 (0.060, 0.264) | | | | | 0.002** | | |
|  |  |  | BPb | | | 0.114 (0.021, 0.208) | | | | | 0.016* | | | 0.116 (0.023, 0.209) | | | | | 0.015* | | |
| BAl, aluminum in blood; BCr, chromium in blood; BCu, cuprum in blood; BZn; zinc in blood; BAs, arsenic in blood; BCd, cadmium in blood; BPb, lead in blood; ALT, alanine aminotransferase; AST, aspartate aminotransferase; TBil, total bilirubin; DBil, direct bilirubin; IBil, indirect bilirubin; ALP, alkaline phosphatase; GGT, gamma glutamyl transpeptidase; CHE, cholinesterase; TBA, total bile acid; β: regression coefficient; CI: confidence interval.  Heavy metals in blood and liver function parameters were log transformed. Coefficients (β) represent the estimated percent change in the liver function index associated with a 1% increase in blood metal concentration.  Linear regression was used to test the relationship between heavy metals in blood and liver function indexes. *β* > 0 means positive correlation, *β* < 0 means negative correlation. Model 1: Not adjustment; Model 2: Adjusted for age, sex, BMI, smoking status, alcohol consumption and tea drinking.  ***P* < 0.01; **P* < 0.05. | | | | | | | | | | | | | | | | | | | | | |
| **Table S5.** The relationship between heavy metals in blood and liver function parameters stratified by gender. | | | | | | | | | | | | | | | | | | | | |  |
| Liver function parameters | | Metals | | | Male | | | | | | | | Female | | | | | | | |  |
|  |  |  |  |  | *β (95% Cl)* | | | | | *P* Value | | | *β (95% Cl)* | | | | | *P* Value | | |  |
| ALT | | BAl | | | 0.075 (-0.076, 0.225) | | | | | 0.328 | | | 0.020 (-0.097, 0.136) | | | | | 0.742 | | |  |
|  |  | BCr | | | -0.124 (-0.283, 0.036) | | | | | 0.127 | | | -0.027 (-0.145, 0.091) | | | | | 0.652 | | |  |
|  |  | BCu | | | 0.075 (-0.082, 0.231) | | | | | 0.348 | | | 0.029 (-0.088, 0.146) | | | | | 0.628 | | |  |
|  |  | BZn | | | 0.008 (-0.155, 0.170) | | | | | 0.926 | | | 0.009 (-0.104, 0.122) | | | | | 0.876 | | |  |
|  |  | BAs | | | 0.039 (-0.128, 0.207) | | | | | 0.642 | | | 0.052 (-0.064, 0.168) | | | | | 0.376 | | |  |
|  |  | BCd | | | 0.076 (-0.117, 0.270) | | | | | 0.174 | | | 0.191 (0.031, 0.350) | | | | | 0.038* | | |  |
|  |  | BPb | | | 0.092 (-0.092, 0.276) | | | | | 0.080 | | | -0.088 (-0.229, 0.053) | | | | | 0.741 | | |  |
| AST | | BAl | | | 0.084 (-0.072, 0.239) | | | | | 0.291 | | | 0.085 (-0.033, 0.204) | | | | | 0.157 | | |  |
|  |  | BCr | | | -0.041 (-0.207, 0.126) | | | | | 0.630 | | | -0.048 (-0.169, 0.072) | | | | | 0.429 | | |  |
|  |  | BCu | | | 0.054 (-0.109, 0.217) | | | | | 0.513 | | | 0.054 (-0.064, 0.173) | | | | | 0.368 | | |  |
|  |  | BZn | | | 0.008 (-0.160, 0.177) | | | | | 0.922 | | | -0.027 (-0.142, 0.088) | | | | | 0.649 | | |  |
|  |  | BAs | | | -0.005 (-0.179, 0.168) | | | | | 0.951 | | | 0.001 (-0.117, 0.120) | | | | | 0.982 | | |  |
|  |  | BCd | | | 0.055 (-0.146, 0.255) | | | | | 0.414 | | | 0.205 (0.043, 0.368) | | | | | 0.041* | | |  |
|  |  | BPb | | | 0.050 (-0.141, 0.242) | | | | | 0.283 | | | -0.128 (-0.272, 0.016) | | | | | 0.875 | | |  |
| TBil | | BAl | | | -0.087 (-0.248, 0.074) | | | | | 0.289 | | | 0.047 (-0.067, 0.161) | | | | | 0.418 | | |  |
|  |  | BCr | | | 0.078 (-0.094, 0.249) | | | | | 0.374 | | | -0.102 (-0.216, 0.012) | | | | | 0.080 | | |  |
|  |  | BCu | | | -0.161 (-0.328, 0.005) | | | | | 0.058 | | | -0.046 (-0.160, 0.068) | | | | | 0.424 | | |  |
|  |  | BZn | | | 0.120 (-0.054, 0.293) | | | | | 0.175 | | | 0.002 (-0.109, 0.112) | | | | | 0.976 | | |  |
|  |  | BAs | | | -0.049 (-0.228, 0.130) | | | | | 0.588 | | | -0.130 (-0.243, -0.018) | | | | | 0.023* | | |  |
|  |  | BCd | | | -0.038 (-0.241, 0.166) | | | | | 0.120 | | | -0.138 (-0.295, 0.019) | | | | | 0.082 | | |  |
|  |  | BPb | | | -0.148 (-0.342, 0.046) | | | | | 0.063 | | | -0.049 (-0.188, 0.091) | | | | | 0.116 | | |  |
| DBil | | BAl | | | -0.098 (-0.259, 0.062) | | | | | 0.228 | | | 0.028 (-0.087, 0.143) | | | | | 0.633 | | |  |
|  |  | BCr | | | -0.145 (-0.315, 0.025) | | | | | 0.093 | | | -0.227 (-0.340, -0.114) | | | | | <0.001** | | |  |
|  |  | BCu | | | -0.132 (-0.299, 0.034) | | | | | 0.118 | | | -0.006 (-0.121, 0.109) | | | | | 0.913 | | |  |
|  |  | BZn | | | 0.230 (0.061, 0.400) | | | | | 0.008** | | | 0.148 (0.038, 0.258) | | | | | 0.009** | | |  |
|  |  | BAs | | | -0.094 (-0.272, 0.084) | | | | | 0.297 | | | -0.150 (-0.263, -0.038) | | | | | 0.009** | | |  |
|  |  | BCd | | | -0.080 (-0.280, 0.120) | | | | | 0.210 | | | -0.088 (-0.243, 0.067) | | | | | 0.102 | | |  |
|  |  | BPb | | | -0.226 (-0.417, -0.036) | | | | | 0.011* | | | -0.235 (-0.392, -0.077) | | | | | 0.001** | | |  |
| IBil | | BAl | | | -0.074 (-0.233, 0.084) | | | | | 0.354 | | | 0.044 (-0.071, 0.159) | | | | | 0.449 | | |  |
|  |  | BCr | | | 0.173 (0.007, 0.340) | | | | | 0.042* | | | -0.021 (-0.138, 0.095) | | | | | 0.718 | | |  |
|  |  | BCu | | | -0.155 (-0.318, 0.009) | | | | | 0.063 | | | -0.057 (-0.172, 0.058) | | | | | 0.332 | | |  |
|  |  | BZn | | | 0.064 (-0.107, 0.235) | | | | | 0.461 | | | -0.050 (-0.162, 0.061) | | | | | 0.377 | | |  |
|  |  | BAs | | | -0.024 (-0.200, 0.151) | | | | | 0.784 | | | -0.093 (-0.208, 0.021) | | | | | 0.108 | | |  |
|  |  | BCd | | | -0.007 (-0.210, 0.197) | | | | | 0.104 | | | -0.235 (-0.392, -0.077) | | | | | 0.001** | | |  |
|  |  | BPb | | | -0.094 (-0.287, 0100) | | | | | 0.184 | | | -0.045 (-0.095, 0.185) | | | | | 0.122 | | |  |
| ALP | | BAl | | | 0.041 (-0.102, 0.185) | | | | | 0.571 | | | -0.043 (-0.161, 0.075) | | | | | 0.472 | | |  |
|  |  | BCr | | | -0.144 (-0.295, 0.008) | | | | | 0.063 | | | 0.083 (-0.036, 0.203) | | | | | 0.171 | | |  |
|  |  | BCu | | | 0.016 (-0.134, 0.166) | | | | | 0.832 | | | 0.099 (-0.019, 0.217) | | | | | 0.099 | | |  |
|  |  | BZn | | | -0.142 (-0.295, 0.011) | | | | | 0.069 | | | 0.101 (-0.013, 0.215) | | | | | 0.083 | | |  |
|  |  | BAs | | | 0.024 (-0.136, 0.183) | | | | | 0.769 | | | 0.043 (-0.075, 0.161) | | | | | 0.471 | | |  |
|  |  | BCd | | | 0.016 (-0.168, 0.201) | | | | | 0.378 | | | 0.086 (-0.076, 0.247) | | | | | 0.077 | | |  |
|  |  | BPb | | | 0.066 (-0.110, 0.241) | | | | | 0.263 | | | 0.059 (-0.084, 0.202) | | | | | 0.103 | | |  |
| GGT | | BAl | | | 0.072 (-0.051, 0.194) | | | | | 0.250 | | | -0.077 (-0.196, 0.042) | | | | | 0.205 | | |  |
|  |  | BCr | | | 0.008 (-0.123, 0.139) | | | | | 0.906 | | | 0.004 (-0.116, 0.125) | | | | | 0.942 | | |  |
|  |  | BCu | | | 0.025 (-0.103, 0.153) | | | | | 0.698 | | | 0.070 (-0.049, 0.189) | | | | | 0.249 | | |  |
|  |  | BZn | | | -0.026 (-0.159, 0.106) | | | | | 0.696 | | | 0.008 (-0.108, 0.124) | | | | | 0.892 | | |  |
|  |  | BAs | | | 0.079 (-0.056, 0.215) | | | | | 0.249 | | | 0.162 (0.044, 0.279) | | | | | 0.007** | | |  |
|  |  | BCd | | | 0.099 (-0.049, 0.247) | | | | | 0.202 | | | 0.272 (0.110, 0.433) | | | | | <0.001** | | |  |
|  |  | BPb | | | 0.192 (0.052, 0.333) | | | | | <0.001** | | | -0.047 (-0.069, 0.254) | | | | | 0.108 | | |  |
| CHE | | BAl | | | 0.091 (-0.043, 0.225) | | | | | 0.180 | | | -0.097 (-0.215, 0.021) | | | | | 0.108 | | |  |
|  |  | BCr | | | -0.045 (-0.188, 0.098) | | | | | 0.536 | | | 0.026 (-0.094, 0.146) | | | | | 0.673 | | |  |
|  |  | BCu | | | 0.046 (-0.095, 0.186) | | | | | 0.520 | | | 0.026 (-0.092, 0.145) | | | | | 0.662 | | |  |
|  |  | BZn | | | 0.089 (-0.056, 0.233) | | | | | 0.227 | | | -0.003 (-0.118, 0.112) | | | | | 0.963 | | |  |
|  |  | BAs | | | 0.137 (-0.011, 0.285) | | | | | 0.068 | | | 0.116 (-0.002, 0.233) | | | | | 0.053 | | |  |
|  |  | BCd | | | 0.049 (-0.219, 0.122) | | | | | 0.737 | | | 0.093 (-0.069, 0.254) | | | | | 0.102 | | |  |
|  |  | BPb | | | 0.141 (-0.021, 0.304) | | | | | 0.111 | | | 0.039 (-0.105, 0.183) | | | | | 0.176 | | |  |
| TBA | | BAl | | | -0.071 (-0.223, 0.080) | | | | | 0.355 | | | 0.045 (-0.073, 0.162) | | | | | 0.452 | | |  |
|  |  | BCr | | | 0.146 (-0.014, 0.305) | | | | | 0.074 | | | 0.041 (-0.078, 0.160) | | | | | 0.498 | | |  |
|  |  | BCu | | | 0.113 (-0.044, 0.270) | | | | | 0.157 | | | -0.049 (-0.167, 0.069) | | | | | 0.413 | | |  |
|  |  | BZn | | | -0.007 (-0.171, 0.157) | | | | | 0.934 | | | -0.097 (-0.210, 0.016) | | | | | 0.093 | | |  |
|  |  | BAs | | | 0.102 (-0.066, 0.269) | | | | | 0.231 | | | 0.043 (-0.074, 0160) | | | | | 0.472 | | |  |
|  |  | BCd | | | 0.164 (-0.030, 0.358) | | | | | 0.076 | | | 0.126 (-0.035, 0.286) | | | | | 0.112 | | |  |
|  |  | BPb | | | 0.006 (-0.179, 0.191) | | | | | 0.189 | | | 0.052 (-0.090, 0.194) | | | | | 0.339 | | |  |
| BAl, aluminum in blood; BCr, chromium in blood; BCu, cuprum in blood; BZn; zinc in blood; BAs, arsenic in blood; BCd, cadmium in blood; BPb, lead in blood; ALT, alanine aminotransferase; AST, aspartate aminotransferase; TBil, total bilirubin; DBil, direct bilirubin; IBil, indirect bilirubin; ALP, alkaline phosphatase; GGT, gamma glutamyl transpeptidase; CHE, cholinesterase; TBA, total bile acid; β: regression coefficient; CI: confidence interval.  Heavy metals in blood and liver function parameters were log transformed. Coefficients (β) represent the estimated percent change in the liver function index associated with a 1% increase in blood metal concentration.  Linear regression was used to test the relationship between heavy metals in blood and liver function indexes. *β* > 0 means positive correlation, *β* < 0 means negative correlation. Model was adjusted for age, BMI, smoking status, alcohol consumption and tea drinking.  ***P* < 0.01; **P* < 0.05. | | | | | | | | | | | | | | | | | | | | |  |
| **Table S6.** PIP values of each heavy metal in blood in the BKMR model. | | | | | | | | | | | | | | | | | | | | |  |
| Metals | | PIP | | | | | | | | | | | | | | | | | | |  |
|  |  | ALT | | | AST | | | TBil | DBil | IBil | | | ALP | | | GGT | CHE | TBA | | |  |
| BAl  BCr | | 0.313 | | | 0.578 | | | 0.178 | 0.420 | 0.190 | | | 0.041 | | | 0.048 | 0.031 | 0.038 | | |  |
|  |  | 0.327 | | | 0.485 | | | 0.157 | 0.991 | 0.140 | | | 0.077 | | | 0.151 | 0.161 | 0.251 | | |  |
| BCu  BZn | | 0.305 | | | 0.481 | | | 0.175 | 0.917 | 0.282 | | | 0.140 | | | 0.088 | 0.064 | 0.158 | | |  |
|  |  | 0.265 | | | 0.387 | | | 0.189 | 1.000 | 0.159 | | | 0.059 | | | 0.099 | 0.095 | 0.172 | | |  |
| BAs | | 0.260 | | | 0.399 | | | 0.787 | 1.000 | 0.289 | | | 0.030 | | | 0.497 | 0.320 | 0.585 | | |  |
| BCd | | 0.742 | | | 0.647 | | | 0.538 | 0.965 | 0.738 | | | 0.137 | | | 0.999 | 0.175 | 0.623 | | |  |
| BPb | | 0.267 | | | 0.449 | | | 0.576 | 0.907 | 0.198 | | | 0.162 | | | 0.097 | 0.128 | 0.412 | | |  |
| PIP, posterior inclusion probability; BAl, aluminum in blood; BCr, chromium in blood; BCu, cuprum in blood; BZn; zinc in blood; BAs, arsenic in blood; BCd, cadmium in blood; BPb, lead in blood; ALT, alanine aminotransferase; AST, aspartate aminotransferase; TBil, total bilirubin; DBil, direct bilirubin; IBil, indirect bilirubin; ALP, alkaline phosphatase; GGT, gamma glutamyl transpeptidase; CHE, cholinesterase; TBA, total bile acid.  Heavy metals in blood and liver function parameters were log transformed. | | | | | | | | | | | | | | | | | | | | |  |

| **Table S7.** Association between WQS regression index of heavy metals in blood and liver function parameters. | | | |
| --- | --- | --- | --- |
| Liver function parameters | *β (95% CI)* | *t* Value | *P* Value |
| ALT | 0.051 (-0.029, 0.132) | 1.249 | 0.213 |
| AST | 0.055 (-0.028, 0.137) | 1.302 | 0.194 |
| TBil | -0.027 (-0.080, 0.027) | -1.010 | 0.313 |
| DBil | 0.030 (-0.022, 0.083) | 1.126 | 0.261 |
| IBil | -0.057 (-0.119, 0.005) | -1.809 | 0.072 |
| ALP | 0.050 (-0.025, 0.125) | 1.306 | 0.193 |
| GGT | 0.105 (0.034, 0.176) | 2.888 | 0.004** |
| CHE | 0.082 (0.006, 0.159) | 2.121 | 0.035* |
| TBA | 0.073 (-0.001, 0.147) | 1.942 | 0.053 |
| ALT, alanine aminotransferase; AST, aspartate aminotransferase; TBil, total bilirubin; DBil, direct bilirubin; IBil, indirect bilirubin; ALP, alkaline phosphatase; GGT, gamma glutamyl transpeptidase; CHE, cholinesterase; TBA, total bile acid; β: regression coefficient; CI: confidence interval.  Heavy metals in blood and liver function parameters were log transformed.  The gWQS model was used to test the relationship between heavy metals mixture and biomarkers. *β* > 0 means positive correlation, *β* < 0 means negative correlation.  Adjusted for age, sex, BMI, smoking status, alcohol consumption and tea drinking.  ***P* < 0.01; **P* < 0.05 | | | |

| **Table S8.** Estimated risk and weighted values of heavy metals in blood for liver function by WQS models. | | | | | |
| --- | --- | --- | --- | --- | --- |
| Liver function parameters | Contribution | Metals | Weight | Percent (%) | Cumulative Percent (%) |
| ALT | 1 | BCd | 4.68×10^-1^ | 46.79% | 46.79% |
|  | 2 | BCu | 1.64×10^-1^ | 16.43% | 63.22% |
|  | 3 | BAl | 1.45×10^-1^ | 14.50% | 77.72% |
|  | 4 | BZn | 1.36×10^-1^ | 13.60% | 91.32% |
|  | 5 | BAs | 5.15×10^-2^ | 5.15% | 96.47% |
|  | 6 | BCr | 2.43×10^-2^ | 2.43% | 98.90% |
|  | 7 | BPb | 1.10×10^-2^ | 1.10% | 100.00% |
| AST | 1 | BCd | 3.94×10^-1^ | 39.37% | 39.37% |
|  | 2 | BCu | 2.27×10^-1^ | 22.73% | 62.10% |
|  | 3 | BAl | 2.10×10^-1^ | 21.00% | 83.10% |
|  | 4 | BZn | 6.74×10^-2^ | 6.74% | 89.84% |
|  | 5 | BAs | 4.81×10^-2^ | 4.81% | 94.64% |
|  | 6 | BCr | 4.64×10^-2^ | 4.64% | 99.29% |
|  | 7 | BPb | 7.15×10^-3^ | 0.71% | 100.00% |
| TBil | 1 | BZn | 7.56×10^-1^ | 75.57% | 75.57% |
|  | 2 | BAs | 1.18×10^-1^ | 11.77% | 87.34% |
|  | 3 | BCd | 7.13×10^-2^ | 7.13% | 94.47% |
|  | 4 | BCu | 2.73×10^-2^ | 2.73% | 97.20% |
|  | 5 | BAl | 2.51×10^-2^ | 2.51% | 99.71% |
|  | 6 | BCr | 2.87×10^-3^ | 0.29% | 100.00% |
|  | 7 | BPb | 3.42×10^-10^ | 0.00% | 100.00% |
| DBil | 1 | BZn | 7.03×10^-1^ | 70.31% | 70.31% |
|  | 2 | BCu | 1.66×10^-1^ | 16.59% | 86.91% |
|  | 3 | BAl | 5.67×10^-2^ | 5.67% | 92.58% |
|  | 4 | BCd | 5.14×10^-2^ | 5.14% | 97.72% |
|  | 5 | BAs | 2.28×10^-2^ | 2.28% | 100.00% |
|  | 6 | BPb | 8.81×10^-11^ | 0.00% | 100.00% |
|  | 7 | BCr | 2.16×10^-11^ | 0.00% | 100.00% |
| IBil | 1 | BZn | 4.81×10^-1^ | 48.14% | 48.14% |
|  | 2 | BAs | 3.11×10^-1^ | 31.14% | 79.28% |
|  | 3 | BCr | 1.16×10^-1^ | 11.60% | 90.88% |
|  | 4 | BAl | 3.55×10^-2^ | 3.55% | 94.42% |
|  | 5 | BCd | 3.13×10^-2^ | 3.13% | 97.56% |
|  | 6 | BPb | 1.34×10^-2^ | 1.34% | 98.89% |
|  | 7 | BCu | 1.11×10^-2^ | 1.11% | 100.00% |
| ALP | 1 | BCd | 2.68×10^-1^ | 26.83% | 26.83% |
|  | 2 | BPb | 2.52×10^-1^ | 25.22% | 52.05% |
|  | 3 | BCu | 1.63×10^-1^ | 16.27% | 68.32% |
|  | 4 | BAl | 1.32×10^-1^ | 13.24% | 81.56% |
|  | 5 | BAs | 1.31×10^-1^ | 13.12% | 94.67% |
|  | 6 | BZn | 4.97×10^-2^ | 4.97% | 99.64% |
|  | 7 | BCr | 3.57×10^-3^ | 0.36% | 100.00% |
| GGT | 1 | BCd | 4.71×10^-1^ | 47.14% | 47.14% |
|  | 2 | BZn | 1.57×10^-1^ | 15.69% | 62.83% |
|  | 3 | BPb | 1.49×10^-1^ | 14.94% | 77.77% |
|  | 4 | BAs | 8.73×10^-2^ | 8.73% | 86.51% |
|  | 5 | BCr | 4.62×10^-2^ | 4.62% | 91.13% |
|  | 6 | BCu | 4.44×10^-2^ | 4.44% | 95.57% |
|  | 7 | BAl | 4.43×10^-2^ | 4.43% | 100.00% |
| CHE | 1 | BAs | 3.25×10^-1^ | 32.53% | 32.53% |
|  | 2 | BCd | 2.14×10^-1^ | 21.37% | 53.89% |
|  | 3 | BAl | 1.70×10^-1^ | 16.98% | 70.87% |
|  | 4 | BPb | 1.43×10^-1^ | 14.33% | 85.20% |
|  | 5 | BCu | 1.19×10^-1^ | 11.95% | 97.15% |
|  | 6 | BZn | 2.50×10^-2^ | 2.50% | 99.65% |
|  | 7 | BCr | 3.51×10^-3^ | 0.35% | 100.00% |
| TBA | 1 | BCd | 3.75×10^-1^ | 37.54% | 37.54% |
|  | 2 | BCr | 3.07×10^-1^ | 30.68% | 68.23% |
|  | 3 | BPb | 1.43×10^-1^ | 14.29% | 82.51% |
|  | 4 | BAl | 8.49×10^-2^ | 8.49% | 91.00% |
|  | 5 | BAs | 4.96×10^-2^ | 4.96% | 95.96% |
|  | 6 | BZn | 3.22×10^-2^ | 3.22% | 99.18% |
|  | 7 | BCu | 8.22×10^-3^ | 0.82% | 100.00% |
| BAl, aluminum in blood; BCr, chromium in blood; BCu, cuprum in blood; BZn; zinc in blood; BAs, arsenic in blood; BCd, cadmium in blood; BPb, lead in blood; ALT, alanine aminotransferase; AST, aspartate aminotransferase; TBil, total bilirubin; DBil, direct bilirubin; IBil, indirect bilirubin; ALP, alkaline phosphatase; GGT, gamma glutamyl transpeptidase; CHE, cholinesterase; TBA, total bile acid.  Heavy metals in blood and liver function indexes were log transformed.  Adjusted for age, sex, BMI, smoking status, alcohol consumption and tea drinking. | | | | | |

| **Table S9.** Association between qgcomp index of heavy metals in blood and liver function parameters. | | | |
| --- | --- | --- | --- |
| Liver function parameters | *β (95% CI)* | *t* Value | *P* Value |
| ALT | 0.081 (0.010, 0.152) | 2.234 | 0.026* |
| AST | 0.065 (-0.008, 0.137) | 1.752 | 0.080 |
| TBil | -0.092 (-0.162, -0.021) | -2.541 | 0.011* |
| DBil | -0.094 (-0.160, -0.027) | -2.754 | 0.006** |
| IBil | -0.087 (-0.158, -0.016) | -2.396 | 0.017* |
| ALP | 0.054 (-0.016, 0.125) | 1.507 | 0.133 |
| GGT | 0.106 (0.039, 0.172) | 3.116 | 0.002** |
| CHE | 0.054 (-0.016, 0.124) | 1.501 | 0.134 |
| TBA | 0.087 (0.017, 0.158) | 2.426 | 0.016* |
| ALT, alanine aminotransferase; AST, aspartate aminotransferase; TBil, total bilirubin; DBil, direct bilirubin; IBil, indirect bilirubin; ALP, alkaline phosphatase; GGT, gamma glutamyl transpeptidase; CHE, cholinesterase; TBA, total bile acid; β: regression coefficient; CI: confidence interval.  Heavy metals in blood and liver function parameters were log transformed.  The qgcomp model was used to test the relationship between heavy metals mixture and biomarkers. *β* > 0 means positive correlation, *β* < 0 means negative correlation.  Adjusted for age, sex, BMI, smoking status, alcohol consumption and tea drinking.  ***P* < 0.01; **P* < 0.05 | | | |

| **Table S10.** Estimated risk and weighted values of heavy metals in blood for liver function by qgcomp model. | | | | |
| --- | --- | --- | --- | --- |
| Liver function parameters | Contribution | Metals | Negative weight | Positive weight |
| ALT | 1 | BCr | 0.693 | - |
|  | 2 | BCd | - | 0.593 |
|  | 3 | BPb | 0.307 | - |
|  | 4 | BCu | - | 0.187 |
|  | 5 | BZn | - | 0.119 |
|  | 6 | BAl | - | 0.070 |
|  | 7 | BAs | - | 0.031 |
| AST | 1 | BCd | - | 0.555 |
|  | 2 | BPb | 0.420 | - |
|  | 3 | BAs | 0.315 | - |
|  | 4 | BCu | - | 0.249 |
|  | 5 | BCr | 0.244 | - |
|  | 6 | BAl | - | 0.197 |
|  | 7 | BZn | 0.021 | - |
| TBil | 1 | BZn | - | 0.784 |
|  | 2 | BCd | 0.395 | - |
|  | 3 | BAs | 0.258 | - |
|  | 4 | BAl | - | 0.216 |
|  | 5 | BCu | 0.140 | - |
|  | 6 | BCr | 0.105 | - |
|  | 7 | BPb | 0.102 | - |
| DBil | 1 | BZn | - | 0.889 |
|  | 2 | BCr | 0.449 | - |
|  | 3 | BAs | 0.223 | - |
|  | 4 | BPb | 0.190 | - |
|  | 5 | BCd | 0.118 | - |
|  | 6 | BCu | - | 0.111 |
|  | 7 | BAl | 0.021 | - |
| IBil | 1 | BCr | - | 0.617 |
|  | 2 | BCd | 0.558 | - |
|  | 3 | BAl | - | 0.282 |
|  | 4 | BCu | 0.246 | - |
|  | 5 | BAs | 0.142 | - |
|  | 6 | BPb | 0.100 | - |
|  | 7 | BZn | 0.054 | - |
| ALP | 1 | BAl | 0.517 | - |
|  | 2 | BCr | 0.402 | - |
|  | 3 | BCu | - | 0.390 |
|  | 4 | BCd | 0.367 | 0.367 |
|  | 5 | BPb | 0.177 | 0.177 |
|  | 6 | BAs | 0.081 | - |
|  | 7 | BZn | - | 0.067 |
| GGT | 1 | BCd | - | 0.661 |
|  | 2 | BCr | 0.473 | - |
|  | 3 | BAl | 0.406 | - |
|  | 4 | BAs | - | 0.229 |
|  | 5 | BZn | 0.121 | - |
|  | 6 | BCu | - | 0.109 |
|  | 7 | BPb | - | 0.001 |
| CHE | 1 | BCr | 0.534 | - |
|  | 2 | BAs |  | 0.470 |
|  | 3 | BAl | 0.325 | - |
|  | 4 | BPb | - | 0.264 |
|  | 5 | BCd | - | 0.237 |
|  | 6 | BCu | 0.140 | - |
|  | 7 | BZn | - | 0.030 |
| TBA | 1 | BZn | 0.645 | - |
|  | 2 | BCd | - | 0.434 |
|  | 3 | BCr | - | 0.320 |
|  | 4 | BPb | 0.215 | - |
|  | 5 | BAs | - | 0.192 |
|  | 6 | BCu | 0.141 | - |
|  | 7 | BAl | - | 0.054 |
| BAl, aluminum in blood; BCr, chromium in blood; BCu, cuprum in blood; BZn; zinc in blood; BAs, arsenic in blood; BCd, cadmium in blood; BPb, lead in blood; ALT, alanine aminotransferase; AST, aspartate aminotransferase; TBil, total bilirubin; DBil, direct bilirubin; IBil, indirect bilirubin; ALP, alkaline phosphatase; GGT, gamma glutamyl transpeptidase; CHE, cholinesterase; TBA, total bile acid.  Heavy metals in blood and liver function parameters were log transformed.  Adjusted for age, sex, BMI, smoking status, alcohol consumption and tea drinking. | | | | |
