# Supplementary material for: Association of heavy metal mixtures with liver function biomarkers: multi-model analysis identifies cadmium as the primary driver
Source: Front Public Health. 2026 Apr 28;14:1817191. doi: 10.3389/fpubh.2026.1817191 (PMC13161090; doi:10.3389/fpubh.2026.1817191)
Supplement: Supplementary file 10 [file Table_6.DOCX]

**Highlights**

- Blood heavy metal mixture was significantly correlated with liver function indices.
- Multi-model consistently highlighted Cd’s predominant role in metal mixtures.
- Hepatic lipid metabolism disorder as a key mechanism of Cd hepatotoxicity.
