# Supplementary material for: Association of heavy metal mixtures with liver function biomarkers: multi-model analysis identifies cadmium as the primary driver
Source: Front Public Health. 2026 Apr 28;14:1817191. doi: 10.3389/fpubh.2026.1817191 (PMC13161090; doi:10.3389/fpubh.2026.1817191)
Supplement: Supplementary file 6 [file Table_2.DOC]

February 25, 2026

Paolo Vineis

Field Chief Editor

*Frontiers in Public Health*

Dear Editor:

I wish to submit an article for publication in ***Frontiers in Public Health****,* titled “Association of heavy metal mixtures with abnormal liver function: Multi-model analysis identifies cadmium as the primary driver.” The paper was coauthored by Honglong Zhang, Xingwang Zhu, Meng Tian, Ruipeng Wang, Tingting Wang, Mingtong Zhang, and Xun Li.

This study was designed to address the current gap in evidence regarding the hepatotoxic effects of co-exposure to environmental heavy metal mixtures in the general middle-aged and elderly population. We employed an innovative integrated approach combining population epidemiology and experimental toxicology. First, within a community-based cohort in Northwest China, we measured multiple blood heavy metals and a comprehensive panel of liver function biomarkers. Advanced statistical models for mixture analysis, including bayesian kernel machine regression (BKMR), weighted quantile sum (WQS) regression, and quantile-based g-computation (qgcomp), were applied to evaluate the combined effects of co-exposure. Subsequently, key epidemiological findings were validated in vivo using a sub-chronic cadmium (Cd) exposure rat model, and potential molecular mechanisms were further explored via hepatic transcriptomic sequencing.

Our research yields several significant and consistent findings: 1) Cd was consistently identified as the primary risk factor for abnormal liver function within the heavy metal mixture across multiple statistical models. 2) Animal experiments confirmed that Cd exposure specifically induces elevated liver injury markers and histopathological alterations. 3) Mechanistically, disruption of hepatic lipid metabolism emerged as a key pathway underlying Cd-induced hepatotoxicity. These results strongly suggest that environmental health risk assessment must shift from the traditional single-metal evaluation towards a mixture-based paradigm.

We believe that this paper will be of interest to the readership of ***Frontiers in Public Health*** because it addresses the pressing public health issue of heavy metal pollution in aging populations. The results of this study provide insights that can inform health management strategies for reducing chronic liver disease risk.

This manuscript has not been published or presented elsewhere in part or in entirety and is not under consideration by another journal. We have read and understood your journal’s policies, and we believe that neither the manuscript nor the study violates any of these. There are no conflicts of interest to declare.

Thank you for your consideration. I look forward to hearing from you.

Sincerely,

Jun Yan

Department of General Surgery, the First Hospital of Lanzhou University

No.1 Donggang West Road, Chengguan District 730030

Lanzhou, Gansu

China

Tel: +86-13919840487

Email: ldyysys@126.com
